# Effectiveness of Interventions for Addressing Digital Exclusion in Older Adults in the Social Care Domain: Rapid Review

**DOI:** 10.2196/70377

**Published:** 2025-12-30

**Authors:** Alesha Wale, Jordan Everitt, Toby Ayres, Chukwudi Okolie, Helen E Morgan, Hannah Shaw, Alison Cooper, Adrian Edwards, Ruth Lewis

**Affiliations:** 1 Public Health Wales NHS Trust Cardiff United Kingdom; 2 Health and Care Research Wales Evidence Centre Cardiff University Cardiff United Kingdom; 3 Health and Care Research Wales Evidence Centre North Wales Medical School Bangor University Bangor United Kingdom

**Keywords:** digital exclusion, digital inclusion, older adults, effectiveness, review, mobile phone

## Abstract

**Background:**

Older adults make up the largest proportion of nonusers of the internet. With the increasing digitalization of services, it is important to identify what interventions are effective at reducing digital exclusion in older adults.

**Objective:**

We aimed to identify what evidence exists on the effectiveness of interventions to address digital exclusion in older adults.

**Methods:**

This rapid review assessed the effectiveness of interventions to address digital exclusion in older adults aged 60 years or older. Searches were conducted in November 2023 across a range of databases and used supplementary search methods. Searches were limited to comparative studies published from 2018 onward in English. Data were analyzed using a narrative synthesis approach.

**Results:**

A total of 21 studies were included that aimed to increase a range of digital literacy skills. Sample sizes ranged from 5 to 381. Intervention approaches varied considerably and were often multicomponent and undertaken in a variety of settings. There is evidence to suggest that a range of interventions can reduce physical, personal, and perceptual barriers and improve older adults’ skills, knowledge, digital literacy, and perceived self-efficacy, reduce technophobia, and increase use of technology. Importantly, findings indicated improvements among a range of subpopulations, including those living in rural areas, at risk of social isolation, who are homebound, of lower socioeconomic groups, and individuals with visual impairment. To achieve improved and sustained digital inclusion in older adults, evidence suggests it may be important to ensure structural barriers, such as access to the internet and affordability of devices, are removed. However, all studies contained methodological limitations and may not be adequately powered to determine effectiveness.

**Conclusions:**

The evidence shows the potential benefits of interventions aimed at improving a range of digital skills and increasing technology use in older adults, which could help to address digital exclusion. The findings of this rapid review can inform the development and delivery of future interventions. However, it is important to consider the context in which the included interventions were used and the lack of certainty of the findings. This review also identified a lack of high-quality evidence, as all studies identified contained methodological limitations and may not have been adequately powered to determine effectiveness. In addition, consideration should also be given to those who do not wish to engage with the online world to ensure they are not left behind.

## Introduction

Digital exclusion refers to the disparity in access to and capability in using information and communications technologies across the population [1]. This leaves certain groups of people unable to fully benefit from digital advancements [2]. This exclusion can be attributed to several factors, including a lack of motivation, where individuals do not perceive the value of being online [3]; issues with accessibility, such as limited physical access to the internet; inability or a deficiency in digital skills; and the inability to afford the necessary technologies and connectivity [4]. The gap between those excluded from and those who can take advantage of digital technologies is known as the “digital divide.” “Digital inclusion” describes the strategies aimed at mitigating barriers to accessing, using, and benefiting from digital tools [5] that can be implemented to reduce the “digital divide.” For the purposes of this rapid review, digital literacy is defined as the capabilities that enable individuals to live, learn, work, and participate fully in a digital society [6].

Digital exclusion of certain population groups can be exacerbated because the contributing factors are often interrelated. For example, older age is associated with disability, deprivation, and poorer digital skills, which are also some of the most significant predictors of digital exclusion [7]. Older adults constitute the largest demographic group of noninternet users [8]. In the United Kingdom, 3.9 million people older than 65 years (representing around 31% of this age group) do not use the internet at home [7]. Of the 2.4 million adults with zero basic digital skills in the United Kingdom, half are aged older than 75 years [9]. Similar findings are reported elsewhere, with lower levels of digital skills being recorded in older adults across Europe [10] and lower rates of internet use being recorded for older adults in the United States [11].

Worldwide, the provision of government services online has been increasing, with over 84% of countries offering at least one online service [12]. As the digitization of health and care services continues, digital exclusion of older adults may also contribute to functional dependence, that is, the requirement of physical assistance to carry out activities online [8]. However, older adults encounter a range of barriers when engaging with digital technologies. These include physical barriers related to age, such as visual impairments and reduced dexterity, as well as personal barriers such as living alone, lower income, and limited digital literacy, or difficulty comprehending digital terminology [13-16]. Perceptual barriers also play a significant role, with negative stereotypes about aging, anxiety about technology use, and a lack of confidence contributing to their exclusion from the digital world [13,15,17,18].

Addressing these challenges is crucial to closing the digital divide and equipping older adults with the skills needed to navigate an increasingly digital society. While many people who do not use the internet cite personal choice as the main reason [9], between 2013 and 2020, the proportion of people older than 75 years using the internet in the United Kingdom almost doubled [19], demonstrating that with the appropriate approach, the digital divide can be closed.

This review was requested by Social Care Wales, which wanted to identify evidence to inform the development of strategies to reduce digital exclusion across Wales. Preliminary work focused on exploring the effectiveness of interventions to support older adults accessing social care services online. However, a lack of research specifically aimed at supporting older adults to access social care services online was identified, so the original scope was broadened to include interventions to support older adults to access and engage with digital technologies for personal use generally. As such, interventions targeting access to health-related services or enhancing work-related digital skills were excluded, as older adults may already be able to access support from health care services or workplaces to develop these skills. This rapid review aimed to examine the effectiveness of interventions aimed at reducing digital exclusion in older adults (defined as those aged 60 years or older). Specifically, we sought to answer the question “What evidence exists on the effectiveness of interventions to address digital exclusion in older adults?”

## Methods

### Study Design

A rapid review methodology was used, which accelerates the process of conducting a conventional systematic review by abbreviating or omitting specific methods to generate the evidence in a resource-efficient manner while still maintaining a similar level of transparency and attention to bias [20]. The rapid review was conducted in line with best practice guidance for rapid reviews [21]. A predefined protocol detailing the eligibility criteria and methods was created and is available on request. The rapid review is structured and reported in line with the PRISMA (Preferred Reporting Items for Systematic Reviews and Meta-Analyses) guidelines for systematic reviews [22].

### Literature Search

Searches were conducted in Social Policy and Practice (Ovid), Scopus, and Sociology Collection. Supplementary searches were conducted in Google Scholar, the Social Care Institute for Evidence, and The King’s Fund. All searches were conducted in November 2023. Secondary sources identified during the preliminary stages of the rapid review were citation-tracked for eligible studies. Searches were limited to include English-language studies. Given the large evidence base identified during the preliminary stages of the rapid review and that digital technologies continuously develop and change, searches were also limited to studies published since 2018 to ensure that findings are most relevant to current technologies that are in use. Search concepts and keywords included digital inclusion, digital exclusion, digital skills, elderly, and older adults. Database search strategies are available in Multimedia Appendix 1. EndNote 20 (Clarivate) was used to manage records and perform deduplication.

### Study Selection Process

Studies were screened for inclusion using the eligibility criteria outlined in Table 1. Studies were screened in Rayyan [23] in a 2-staged process where titles and abstracts were screened for inclusion, followed by the full texts. The process was conducted by 2 independent reviewers, and any disagreements were discussed and resolved within the review team.

**Table 1:** Eligibility criteria.

	Inclusion criteria	Exclusion criteria
Participants	Older adults aged 60 years or older as defined by the Older People’s Commissioner for Wales [24] Caregivers of older adults aged 60 years or older	Adults aged younger than 60 years of age Children
Settings	Any setting except health care	Health care settings (or digitalized health care services)
Intervention or exposure	Interventions to address digital exclusion (in relation to motivation, accessibility [internet], ability and skills, or affordability)	Interventions to assess telemedicine and interventions to improve health literacy
Comparison	No intervention or alternative interventions	No comparator
Outcomes	Any outcomes associated with the measurement of digital inclusivity, including aspects relating to motivation, access, ability, or affordability, for example: Willingness to use technology Digital literacy skills Technology use Ability to use technology Perceptions on the use of technology Level of access to technology Technology knowledge Self-efficacy in using technology Perceptions of the interventions Cost-effectiveness of the intervention	Any other outcomes
Study design	Any comparative study	Noncomparative studies
Countries	All countries	Not applicable
Language of publication	English	Non-English
Publication date	2018 to 2023	Pre-2018
Publication type	Published	Commentaries, editorials, letters, conference abstracts, and preprints

### Data Extraction

For each included study, the citation, study design, details about the intervention, comparator, study aim, data collection methods and dates, outcomes reported, sample size, participants, setting, and key findings were extracted by 1 reviewer and were consistency checked by another.

### Quality Appraisal

Quality appraisal was conducted in duplicate by 2 independent reviewers; any disagreements were discussed and resolved within the review team. The Joanna Briggs Institute quality appraisal checklist for randomized controlled trials [25] and quasi-experimental studies [26] was used to assess the methodological quality of the included studies.

### Synthesis

Due to the heterogeneity of included studies, meta-analysis was not appropriate. Consequently, the evidence was synthesized narratively by outcome to describe the effectiveness of interventions to address digital exclusion in older adults.

## Results

### Literature Search and Study Selection

**Figure 1:**
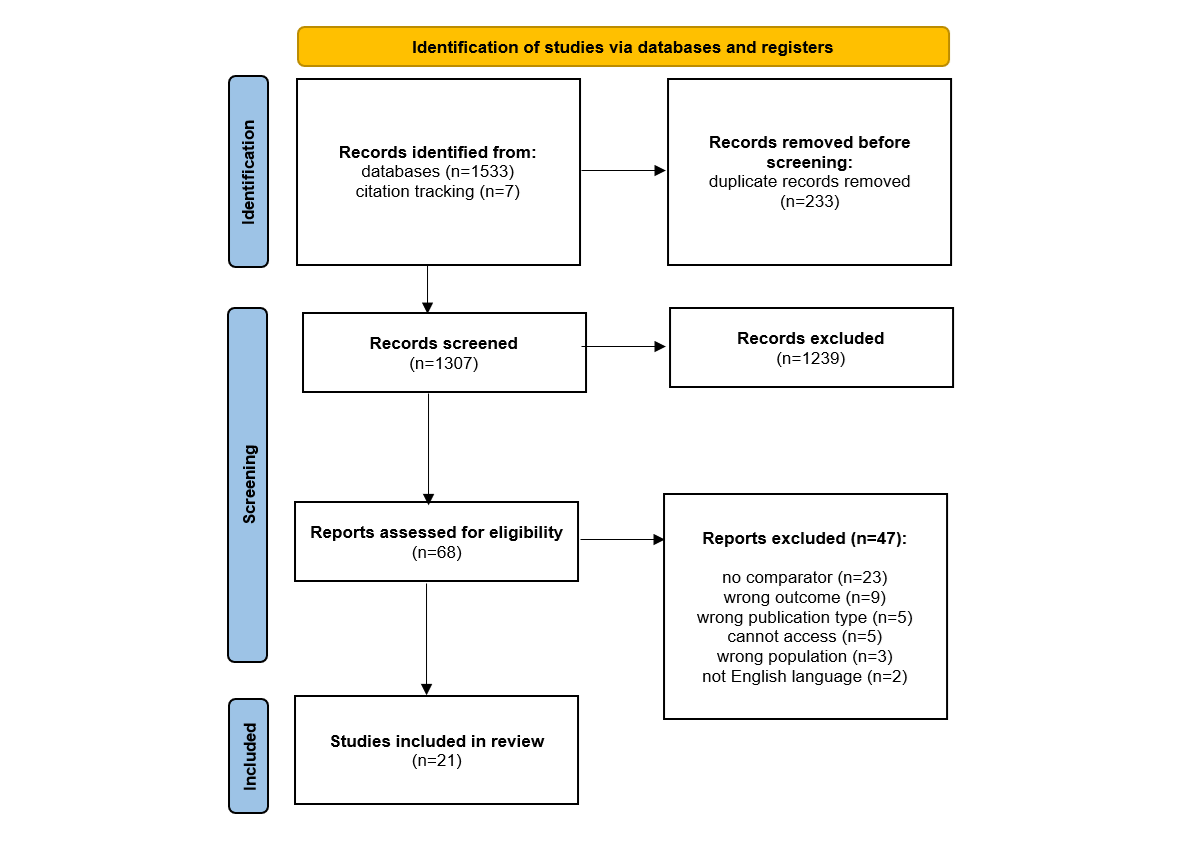
PRISMA flow diagram. PRISMA: Preferred Reporting Items for Systematic Reviews and Meta-Analyses.

A total of 1540 records were identified through searches and citation tracking. After deduplication, 1307 records were screened at title and abstract; of these, 68 were screened at full text, which resulted in 21 studies being included in the review. This study selection process can be seen in Figure 1.

### Quality Appraisal

The 21 studies included 3 randomized controlled trials [27-29], 10 nonrandomized controlled studies [30-39], and 8 uncontrolled before-and-after studies [40-47]. Quality appraisal highlighted methodological limitations within the included studies. The randomized controlled trials were all determined to be of moderate quality; however, they were limited by insufficient reporting on concealed allocation of participants and loss to follow-up as well as uncertainty regarding the reliability of outcome measures used. The nonrandomized controlled trials were determined to be of low quality, and the majority were limited by a lack of between-group comparisons. The uncontrolled before-and-after studies were also determined to be of low quality and were often limited by a lack of certainty regarding the reliability of outcome measures. More details about the quality appraisal results can be seen in Multimedia Appendix 2.

### Study Characteristics

The included studies were published between 2018 and 2023, and were conducted in United States (n=6), South Korea (n=3), Canada (n=2), Mexico (n=2), Australia (n=1), China (n=1), Netherlands (n=1), Peru (n=1), Portugal (n=1), Singapore (n=1), and Spain (n=1). One study was conducted across multiple countries, including the United Kingdom, Latvia, Poland, and Portugal. Eleven of the included studies focused on specific subpopulations within the older adult population, such as adults from small or rural towns (n=4), older adults of low socioeconomic status (n=3), older adults at risk of social isolation (n=2), older adults who are homebound (n=1), and older adults with visual impairment (n=1).

**Figure 2:**
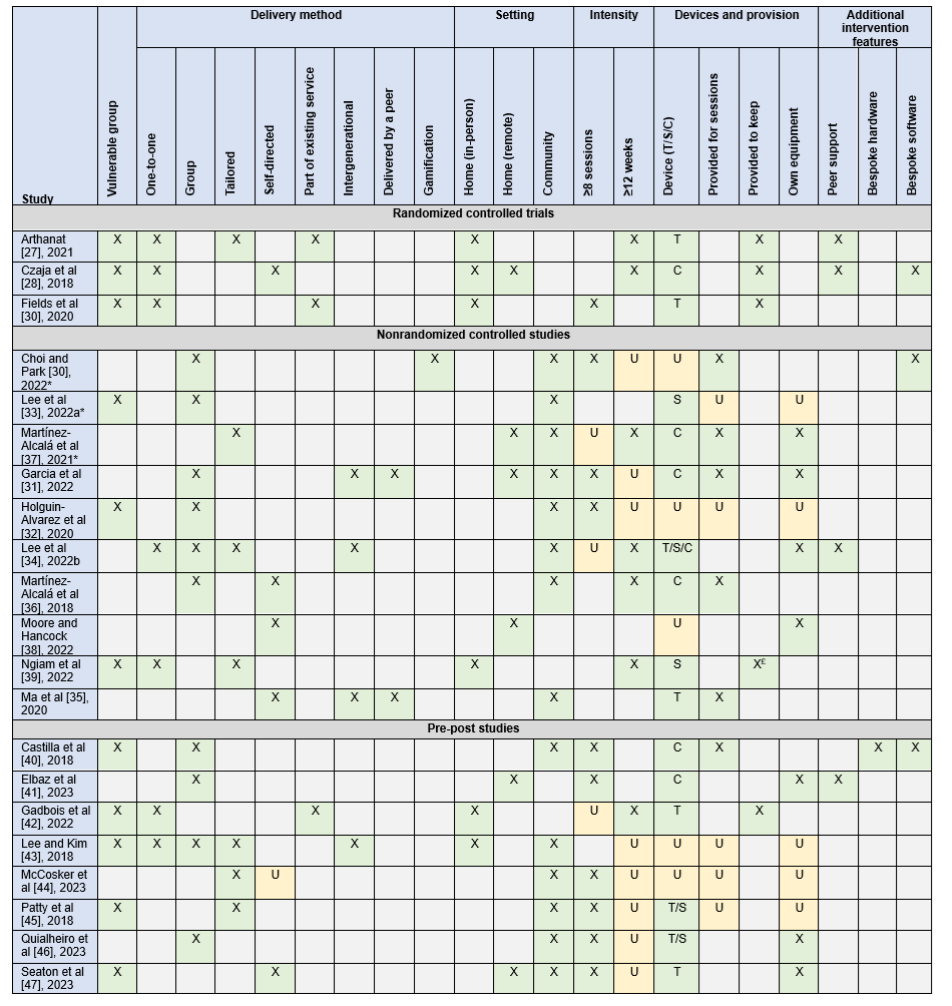
Interventions matrix [27-47]. *Controlled studies that did not provide between group comparisons. ₤: at a cost; C: computer; S: smartphone; T: tablet; U: unclear.

A summary of the study characteristics can be seen in Table 2. Many of the studies contained complex multicomponent interventions and often had overlapping features, which are outlined in Figure 2. As such, the findings were synthesized by outcome rather than intervention type. The included studies all aimed to increase a range of digital literacy skills, which may help to reduce digital exclusion. Some of the interventions included components that addressed aspects relating to people’s ability to use digital technology, by not only addressing their lack of skills, but also targeting potential physical barriers, such as poor eyesight. Some interventions included components that addressed affordability through the provision of free equipment, and some included components relating to motivation, for example, by trying to improve people’s confidence or perceived self-efficacy to use technology, or by demonstrating the usefulness of technology. Additionally, 4 studies included an intergenerational component. Three studies incorporated interventions into existing services, such as a home-delivered meals program for homebound older adults, an occupational therapy program, or a volunteer-based program that provides home visits for older adults experiencing loneliness. Two studies created tailored computer software, 1 study incorporated an online game, and 1 study specifically aimed to teach participants to detect online deception. A detailed description of the interventions can be seen in Multimedia Appendix 3, and the full data extraction table can be seen in Multimedia Appendix 4 [27-47].

Intervention delivery approaches varied; most included studies specified that the interventions were delivered either in the home or online, and either in groups or on a one-to-one basis. Outcomes included digital technology use, digital literacy, participant perceptions of technology use, acceptability of the interventions, and cost-effectiveness. The varying approaches and intervention types used across the included studies, in addition to a lack of standardized definitions for outcomes and inconsistent measurement tools used, made it difficult to further categorize the interventions for the synthesis. The findings are presented according to the outcomes, and the different intervention delivery approaches used are highlighted throughout the results.

**Table 2:** Summary of study characteristics.

Study, year, and country			Intervention details		Participants and setting		Key findings
**Randomized controlled trials (n=3)**							
	Arthanat [27], 2021, United States	Intervention: the i-CHATT^a^ program was an individualized intergenerational Information and Communication Technologies training program for older adults in rural areas. Comparator or control: no intervention was recorded. Duration: 3 months.		Setting: home-based Participants: 97 adults (aged 65+ years)		Technology use: the intervention and control group reported similar rates of technology use at 6 months follow-up; however, the intervention group maintained a more increasing trend during the remaining follow-up points. The group X time interaction for the overall range of technology use was found to be statistically significant (F4,1=2.5; *P*=.04). Self-reported independence: the training group maintained a higher trend in ratings over the control group following the intervention. Technology acceptance: the intervention group (compared to those in the control) expressed that “technology experiences are satisfying” (F4,1=3.5; *P*=.007), “technologies are encouraging” (F4,1=2.4; *P*=.05), “I’m comfortable with technology (F4,1=3.6; *P*=.009), and “I feel good around technology” (F4,1=2.3; *P*=.05).	
	Czaja et al [28], 2018, United States	Intervention: a PRISM^b^ that was for older adults at risk of social isolation. Comparator or control: provision of a notebook with content similar to that in the intervention. Duration: 12 months.		Setting: home-based Participants: 300 adults (aged 65+ years)		Computer comfort: intervention participants significantly improved at 6 and 12 months (b=–1.68; b=–2.32; *P*<.001). Computer interest: intervention participants significantly improved at 6 months (b=–1.52; *P*<.001) and 12 months (b=–0.99; *P*<.01). Computer efficacy: intervention participants significantly improved at 6 months (b=–1.29; *P*<.001) and 12 months (b=–0.94, *P*<.02). Computer proficiency: intervention participants significantly improved at 6 (b=–6.37; *P*<.001) and 12 months (b=–7.06; *P*<.001). Technology acceptance: 123 (82%) intervention participants found PRISM useful in their daily life, 120 (80%) said it made their life easier, 126 (84%) said it improved their daily life, and 109.5 (73%) said it enabled them to accomplish tasks more quickly, 132 (88%) found PRISM easy to use and 120 (80%) felt it was easy to become skilled at using PRISM.	
	Fields et al [29], 2020, United States	Intervention: Tech Allies held one-to-one digital training sessions for isolated older adults. Comparator or control: a waitlist group was used. Duration: 2 months.		Setting: home-based Participants: 83 adults (65+ years or 60+ years with a disability)		Technology use: there were significant improvements in technology use from baseline to 2 months within the intervention group (33% vs 0%; *P*=.004). There was no change over time within the control group (53% vs 60%; *P*=.63). Confidence: there were nonsignificant improvements in confidence in digital skills at 2 months (52% little to no confidence vs 35%; *P*=.13) and no change in the control group (76% vs 77%; *P*≥.99).	
**Nonrandomized controlled trials (n=10)**							
	Choi and Park [30], 2022, South Korea	Intervention: an educational program that combines a decision tree with a game. Comparator or control: general internet and digital device use education was recorded. A duration that was not stated.		Setting: education center Participants: 42 adults (aged 60+ years)		Digital literacy: digital literacy significantly improved in the intervention group (recognition: mean 2.45, SD 0.55, to mean 3.02, SD 0.64; z=–3.45; *P*=.01; behavior: mean 3.16, SD 0.66, to mean 3.67, SD 0.59; z=–2.17; *P*=.001). The digital literacy capability was improved in the comparative group, but it was not statistically significant (recognition: mean 2.34, SD 0.47, to mean 2.51, SD 0.35; z=–1.69; *P*=.10; behavior: mean 3.1, SD 0.81, to mean 3.32, SD 0.34; z=–0.94; *P*=.08). Satisfaction: the average satisfaction of the intervention participants was 4.13 (SD 0.65). Male satisfaction (mean 4.15, SD 0.67) was higher than women's (mean 3.98, SD 0.77). In addition, the average satisfaction score of subjects aged 60 to 65 years was 4.23 (SD 0.59), and for those aged 65 to 69 years were lower at 3.58 (SD 0.85).	
	Garcia et al [31], 2022, multiple countries	Intervention: the Erasmus+ project ICTskills4All examined 3 educational approaches (intergenerational, peer-to-peer, and online). Comparator or control: see above. A duration that was not stated.		Setting: classroom-based and virtual (online group) Participants: 39 adults (55 years)		Information and digital literacy: significant improvements were reported for all groups, peer-to-peer (pre median 2.09, IQR 1.50-3.28 to post median 3.72, IQR 3.34-4.27; *P*<.001), intergenerational (pre median 2.41, IQR 1.65-3.32 to post median 3.91, IQR 3.62-4.26; *P*=.001), and online (pre median 4.13, IQR 3.65-4.37 to post median 4.83, IQR 4.43-4.89; *P*=.02). Communication and collaboration: significant improvements were reported for all groups, Peer-to-peer (pre median 1.78, IQR 1.22-2.94 to post median 3.22, IQR 2.56-3.81; *P*<.001), intergenerational (pre median 2.06, IQR 1.06-3.17 to post median 3.44, IQR 2.81-4; *P*=.01) and online (pre median 3.67 IQR 3-4.11 to post median 4.78, IQR 3.67-4.78; *P*=.05). Comparisons between groups: for peer-to-peer versus intergenerational, no significant differences were found for any outcome. For peer-to-peer versus online, differences in information and digital literacy, communication and collaboration appear to be in favor of peer-to-peer but are not clearly reported. For Intergenerational versus online, differences in information and digital literacy, communication, and collaboration appear to be in favor of the intergenerational, but are not clearly reported.	
	Holguin-Alvarez et al [32], 2020, Peru	Intervention: it was social media program designed to increase digital skills in communities in vulnerable contexts (lower SES^c^). Comparator or control: no intervention was recorded. A duration that was not stated.		Setting: older adult center Participants: 40 adults (81-92 years)		Digital competencies: the intervention group reported greater improvements after the intervention when compared to the control group (t34=–1.264; *P*<.001).	
	Lee et al [33], 2022, South Korea	Intervention: digital literacy education program to improve smartphone usage competency of those living in rural areas. Comparator or control: no intervention was recorded. Duration: 6 weeks.		Setting: 5 locations including Yonsei University Healthy City Research Center, the Wonju Senior Center, and small libraries Participants: 144 adults (aged 65+ years)		Technology use: the frequency of phone calls made using smartphones by the intervention group increased significantly by 8.5% after education (40/63 at baseline vs 37/45 at endline; *t*=1.934; *P*=.03). In contrast, no changes were observed in the control group. No significant increase in taking photos was observed in either group (intervention group: 47/62 vs 40/45; *P*=.09; control group: 46/62 vs 22/36; *P*=.18). A significant increase in ability to video record using a smartphone was found in the intervention group (23/62 vs 26/45; *P*=.049), no improvement was shown for the control group (27/62 vs 13/36; *P*=.53). Self-efficacy: the intervention group improved after the intervention but not significantly (mean 57.1, SD 9.5, to mean 58.1, SD 7.5; *P*=.53), the control group increased significantly (mean 55.6, SD 8.5 to mean 60.1, SD 11.2; *P*=.03). However, when compared no significant difference was reported between groups (*t*=–1.382; *P*=.17).	
	Lee et al [34], 2022, South Korea	Intervention: the Intergenerational Forum is an educational program providing guided instruction and intergenerational exchange. Comparator or control: no intervention was recorded. Duration: 12 weeks.		Setting: 2 large senior centers Participants: 104 adults (aged 65+ years)		eHealth literacy: intervention participants significantly improved after the intervention (t49=−4.23; *P*<.001). Technophobia: interventions participants’ anxiety toward technology was significantly reduced (t49=−2.77; *P*<.01) and confidence in using technology significantly increased (t49=−5.05; *P*<.001) after the intervention. Perceptions of technology: interventions’ participants perceived greater usefulness of the internet (t49=−3.50; *P*<.001). Comparison with control group: the results of ANCOVA indicated that there was no statistically significant difference between the grouping conditions on the adjusted posttest means at the *P*<.05 level for all variables, except for confidence (F1,101=9.99; *P*<.05).	
	Ma et al [35], 2020, China	Intervention: video tutorial-based intervention to enhance technology acceptance. Comparator or control: 3 intervention groups with differing models in the videos (a child, young adult, or older adult). A duration that was not stated.		Setting: senior citizen center Participants: 59 adults (aged 60+ years)		Self-efficacy: self-efficacy significantly increased (mean –1.922, SD 1.640; *t*=–9.002; *P*<.001) after the intervention for all intervention groups. However, the older adult behavior model was more effective than the young or child behavior models in increasing self-efficacy.	
	Martínez-Alcalá et al [36], 2018, Mexico	Intervention: digital literacy workshop delivered on a learning management system. Comparator or control: comparisons within and between the face-to-face and blended delivery models. Duration: 4 months.		Setting: classrooms or a learning system Participants: 98 adults (aged 60+ years)		Digital competence: significantly improved in both delivery methods assessed (z=−6.79; z=−5.30; *P*<.001, for the face-to-face and the blended groups, respectively). Digital literacy evaluation: participants in the blended workshop group reported a significantly greater improvement in SDLE^d^ scores compared to the face-to-face group (U61,37=810.5; *P*<.01). Ease of use: 13 of the older adults indicated a positive agreement, stating that the interaction with the system is clear and understandable, and even the menu is easy to use. Attitude and perceived usefulness: 15 older adults were enthusiastic about using the platform, and 16 participants stated that it is useful and indispensable to implement this type of workshop so that the population acquires digital literacy skills.	
	Martínez-Alcalá et al [37], 2021, Mexico	Intervention: digital literacy program delivered either as a blended, transition (part digital) or fully digital format. Comparator or control: comparisons within and between the different delivery models. Duration: 3-4 months.		Setting: classroom or remote Participants: 20 adults (aged around 60 years)		Digital literacy: statistically significant increases were reported after the intervention regardless of how it was delivered (group 1: blended *t*=6.87; *P*<.001; transition *t*=4.95; *P*<.001; digital *t*=8.92; *P*<.001; group 2: blended *t*=11.57; *P*<.001; transition *t*=9.91; *P*<.001; digital *t*=11.71; *P*<.001).	
	Moore and Hancock [38], 2022, United States	Intervention: it was the MediaWise for Seniors online intervention. Comparator or control: no intervention was recorded. A duration that was not specified (self-directed).		Setting: online Participants: 381 adults (aged 60+ years)		Online deception detection: there was a significant improvement in the ability to judge the veracity of news headlines among the intervention group after the intervention compared to the control group (B=1.073; SE=0.159; *P*<.001). Intervention participants significantly improved their likelihood of accurately discerning fake from true news from 64% (91.5 of 143 participants) to 85% (121.6 of 143 participants) after the intervention. In contrast, control group participants did not significantly improve (55%; 130.9 of 238 participants to 57%; 135.7 of 238 participants). Intervention participants' probability of researching to inform their headline judgments rose from 4% (5.7 of 143 participants) to 71% (101.5 of 143 participants) after the intervention; however, this pattern was not observed in the control group.	
	Ngiam et al [39], 2022, Singapore	Intervention: it was Project Wire Up, a digital literacy program for older adults in vulnerable contexts (low SES). Comparator or control: no intervention was recorded. Duration: typically 3 months.		Setting: home-based Participants: 138 adults (aged 55+ years)		Digital literacy: the intervention group showed a statistically significant change in their mean digital literacy score before and after the program compared to those in the control group (mean difference 2.28; *P*<.001). Through multiple linear regression analyses, this change in digital literacy scores remained independently associated with group membership after adjusting for baseline digital literacy scores and differences in age, gender, education, living arrangement, housing type, and baseline social connectivity and loneliness status (model 2: β=1.91; *P*<.001 and model 3: β=1.90; *P*<.001).	
**Uncontrolled before-and-after studies (n=8)**							
	Castilla et al [40], 2018, Spain	Intervention: Butler 2.0 was an online social network as a digital literacy method for the older adult in rural areas. Comparator or control: baseline measures were recorded. Duration: 8 weeks.		Setting: older adult leisure center Participants: 46 adults (aged 60-76 years)		Interest in using new technologies: statistically significant improvements in participants were reported after the intervention (t45=–3.083; *P*=.003); however, improvements in how participants felt when using new technologies were not statistically significant (t45=–1.4; *P*=.17). Perceived capability to use new technologies: statistically significant improvements were reported after the intervention (t45=–2.613; *P*=.01).	
	Elbaz et al [41], 2023, Canada	Intervention: digital literacy intervention program. Comparator or control: baseline measures were recorded. Duration: 4 weeks.		Setting: online via Zoom (Zoom Communications, Inc) Participants: 5 adults (aged 65+ years)		Computer proficiency: statistically significant improvements were reported after the intervention (mean 17.72, SD 1.94 vs mean 13.24, SD 2.40; t4=−8.910; *P*<.001), mean computer proficiency scores were also significantly higher after the intervention for the computer basics (mean 3.97, SD 0.45 vs mean 3.23, SD 0.60); t4=−5.880; *P*=.004), communication (mean 3.36, SD 0.38 vs mean 2.40, SD 0.48); t4=−8.353; *P*=.001), and internet subscales (mean 3.63, SD 0.16 vs mean 2.71, SD 0.48; t4=−4.257; *P*=.01).	
	Gadbois et al [42], 2022, United States	Intervention: the Talking Tech intervention was embedded within and delivered by a home-delivered meals program. Comparator or control: baseline measures was recorded. Duration: 14 weeks.		Setting: home-based Participants: 21 homebound adults (aged 60+ years)		Technology use: there was a trend toward some increased use of technology after the intervention, but none of the results were statistically significant. Sent email or text messages most days in past month (14/18 vs 13/18; *P*≥.99). Used internet in past month (11/18 vs 13/18; *P*=.27). Online activity engagement score (1.44/18 vs 1.89/18; *P*=.44). Seven (38.89%) participants reported greater use of the internet for activities including shopping, prescriptions, social media, and health-related activities, while 3 (16.67%) reported less use and 8 (44.44%) stayed the same.	
	Lee and Kim [43], 2018, United States	Intervention: it was the IMU^e^ program. Comparator or control: baseline measures were recorded. A duration that was not stated.		Setting: senior centers and housing facilities Participants: 55 adults (aged 65+ years)		eHealth literacy: eHEALS^f^ scores showed significant improvement after the intervention (t54=–5.89; *P*<.001). Computer interest: significant improvements were found in participants' interest in using computers or the internet (t54=–9.24; *P*<.001). Self-efficacy: significant improvements were reported after the intervention (t54=–8.36; *P*<.001). Technophobia: after the intervention, participants' confidence about their skills in using computers or the internet significantly increased (t54=–3.69; *P*<.001) and their anxiety toward technology significantly decreased (t54=2.65; *P*<.01).	
	McCosker et al [44], 2023, Australia	Intervention: national digital inclusion program (Be Connected). Comparator or control: baseline measures were recorded. A duration that was not stated.		Setting: online and face-to-face, community based Participants: 337 adults (aged 50-94 years)		Operational skills: significantly improved in emerging and evolving learners (MD^g^=0.45 and MD=0.26, respectively; *P*<.001) but not for the accomplished learner group (MD=0.04; *P*=.51). Technical confidence: significantly improved in emerging learners (MD=0.83; *P*<.001) and evolving learners (MD=0.37; *P*=.002) but not for the accomplished learner group (MD=0.11; *P*=.26).	
	Patty et al [45], 2018, The Netherlands	Intervention: information and communication technology training for adults with visual impairment. Comparator or control: baseline measures were recorded. Duration: training was tailored to each individual’s needs, which meant training durations varied between participants.		Setting: unclear Participants: 45 adults (mean age 63, SD 16 years)		Digital or information and communication technology skills: the mean D-AI^h^ score (information and communication technology skills) decreased (improved) by 10 points at 3 months follow-up (22.98 vs 12.97; *P*<.01). Cost-effectiveness: the intervention appears to be cost-effective under the assumption that the effects of the training on well-being remain constant for 5 or 10 years. Assuming these effects remain constant for 10 years, this would result in an ICER^i^ of €11,000 (a currency exchange rate of EURO €1=US $1.18 was applicable) per QALY^j^ and €8000 per year of well-being gained, when only the costs of the training are considered. When the total costs of medical consumption are included, the ICER increases to €17,000 per QALY gained and €12,000 per year of well-being gained. Furthermore, when the willingness-to-pay threshold is €20,000 per year of well-being, the probability that the training will be cost-effective is 75% (91% when including only the costs of the training).	
	Quialheiro et al [46], 2023, Portugal	Intervention: it was the OITO^k^ project. Comparator or control: baseline measures were recorded. A duration that was not stated.		Setting: locations varied based on specific project partners Participants: 87 adults (aged 55+ years)		Mobile device proficiency: significant improvements in digital literacy (assessed using the Mobile Device Proficiency Questionnaire) were reported after the intervention and at 1-month follow-up compared to baseline (no *P* value given). Self-reported autonomy: a significant improvement in self-reported autonomy was observed at 1-month follow-up compared with baseline, increasing from 4.5 to 6.7 points, with a score range from 0 to 10 (t40=–7.3; *P*<.001).	
	Seaton et al [47], 2023, Canada	Intervention: it was the Gluu Essentials digital skills training program for those in rural and urban communities. Comparator or control: baseline measures were recorded. Duration: no set duration, providers were free to deliver the program however it worked best for participants and staff.		Setting: self-directed; however, some organizations delivered in-person support in groups Participants: 264 adults (aged 50+ years)		Digital technology use: significant increases were reported for the frequency of going online for shopping (*P*=.01) and accessing government services (*P*=.02), whereas the frequency of going online for email (*P*=.47), banking (*P*=.10), information (*P*=.96), and emergency services (*P*=.42) did not change significantly. Mobile device proficiency: mobile device proficiency improved significantly from baseline to follow-up (MDPQ^l^ total score: 3.93 vs 4.13; t143=4.46; *P*<.001).	

^a^I-CHATT: Individualised Community and Home-Based Access to Technology Training.

^b^PRISM: Personal Reminder System.

^c^SES: socioeconomic status.

^d^SDLE: Senior Digital Literacy Evaluation.

^e^IMU: Intergenerational Mentor-Up.

^f^eHEALS: eHealth Literacy Scale.

^g^MD: mean difference.

^h^D-AI: Dutch Activity Inventory.

^i^ICER: incremental cost-effectiveness ratio.

^j^QALY: quality-adjusted life-year.

^k^OITO: Oficinas de Inclusão Tecnológica Online: Workshops for Online Technological Inclusion.

^l^MDPQ: Mobile Device Proficiency Questionnaire.

### Digital Technology Use

Changes in digital technology use in older adults were reported in 5 studies [27,29,33,42,47], 3 of which incorporated interventions into existing services [27,29,42].

Various forms of technology use were assessed, for example, smartphone use, online banking, shopping, etc. Although findings were mixed, they were generally in favor of the interventions, with some increases in digital technology use found in all studies. A statistically significant increase in all types of technology use assessed at 2 months follow-up compared to a control group was reported in 1 study [29]. However, the results were mixed in 2 studies with statistically significant increases in technology use after the intervention for some activities (eg, going online for shopping or making calls using smartphones) but no significant increases for other activities (eg, sending emails or taking photos) [33,47]. No between-group differences were reported by 1 study; however, no changes were observed in the control group [33]. In the remaining 2 studies, a trend toward increased technology use was identified; the increases were not statistically significant but remained 1 week after the intervention in 1 study [42] and up to 2 years of follow-up in the other when compared to a control group [27].

Increases in digital technology use were also found within subpopulations of older adults, such as those living in rural areas [27,33,47], those who are socially isolated [29], or those who are homebound [42].

### Digital Literacy

The impact of interventions on digital literacy outcomes was assessed in 15 studies [28,30-32,34,36-39,41,43-47]. Various approaches were incorporated, including gamification [30], tailored computer software [27], and intergenerational approaches [31,34,43]. All studies reported some improvements in digital literacy outcomes.

Statistically significant improvements in digital literacy as a standalone outcome were reported after the intervention in 3 studies [30,37,39]. Two of which reported within-group differences, finding digital literacy was also improved in the control or comparator groups [30,37]; however, these improvements were only statistically significant in 1 study [30]. Whereas between-group differences were reported by 1 study and found statistically significant improvements in digital literacy when compared to the control group [39].

The impact of interventions on several proxy outcomes for digital literacy was assessed in 10 studies [28,31,32,36,38,41,44-47]. Digital or information and communication technology skills were found to improve after the intervention in 2 studies reporting statistically significant findings [31,45] and were reported to continue to improve at 3 months follow-up in 1 study [45]. In addition, when comparing delivery methods, 1 study reported that while all groups improved their digital or information and communication technology skills, a peer-to-peer delivery method or an intergenerational approach led to a statistically significant increase when compared to an online approach [31]. When comparing different learner groups, 1 study found that emerging learners and evolving learners significantly improved their operational skills after the intervention, whereas accomplished learners did not improve significantly [44].

eHealth literacy significantly improved after the intervention in 2 studies [34,43]. Statistically significant improvements in computer proficiency were also reported in 2 studies, either directly after the intervention [41] or when compared to a control group at 6 and 12 months follow-up [28]. Statistically significant improvements in mobile device proficiency were reported by 2 studies after the intervention [47] or at 1-month follow-up [46]. Improvements in digital competence were reported after the intervention by 2 studies [32,36]. One study found a statistically significant improvement in digital competence when compared to a control group [32], and the other found that those in the blended workshop groups reported statistically significant improvements when compared to a face-to-face group [36]. Lastly, statistically significant improvements in online deception detection when compared to a control group were reported in 1 study [38].

Improvements in digital literacy and digital literacy proxies within subpopulations of older adults, including those at risk of social isolation [28], lower socioeconomic groups [32,39], those living in rural areas [47], and older adults with visual impairment [45] were also reported.

### Participants’ Perceptions of Technology Use

The impact of interventions on participants’ perceptions of technology use was assessed in 10 studies [27-29,33-35,40,43,44,46]. Some of which incorporated tailored computer software [28,40], used an intergenerational approach [34,35,43], or were incorporated into existing services [29].

Improvements in participants’ acceptability of technology were reported in 4 studies [27,28,40,43]. Statistically significant improvements in computer interest were reported in 2 studies [28,43], either after the intervention [43] or at 12 months follow-up when compared to a control group [28]. Statistically significant improvements in participants’ interest in using new technologies were found in 1 study after the intervention; however, improvements reported in how participants felt when using new technologies were not statistically significant [40]. Statistically significant increases in comfort using technology and feeling good around technology, as well as increases in perceiving technology to be satisfying and encouraging, were found in 1 study when compared to the control group and at a 2-year follow-up [27]. Statistically significant improvements were also reported in computer comfort in 1 study when compared to a control group and remained at 12 months follow-up [28].

Improvements in perceived self-efficacy were reported in 4 studies [28,33,35,43]. These improvements were statistically significant in 2 studies after the intervention [43], or when compared to a control group at 6 and 12 months follow-up [28]. However, no significant difference in self-efficacy between the intervention and control group was reported in 1 study [33]. When different delivery approaches were compared, using an older adult in the tutorials resulted in a statistically significant increase in self-efficacy compared to intergenerational models [35].

A statistically significant reduction in technophobia (assessing confidence and anxiety) after the intervention was reported in 2 studies, both of which used an intergenerational component [34,43]. However, when compared to a control group in 1 study, only confidence was seen to statistically and significantly increase [34].

Statistically significant improvements in participant perceptions of their capability to use new technologies were reported in 1 study after receiving the intervention [40]. Statistically significant improvements in self-reported autonomy were reported in 1 study 1 month after the intervention [46], and higher levels of self-confidence 2 months after the intervention compared to a control group were reported by another study; however, this improvement was not statistically significant [29]. When comparing learner groups, emerging learners and evolving learners significantly improved their technical confidence after the intervention, whereas accomplished learners did not show a significant change [44].

Improvements within subpopulations of older adults, including those living in rural areas [27,40], and those who are socially isolated or are at risk of social isolation [29], were also found.

### Acceptability of Interventions

Participants’ acceptability of the interventions was reported in 9 studies [28-30,36,40-43,47]. Some of which incorporated tailored computer software [28,40], were incorporated into existing services [29,42], used an intergenerational approach [43], or incorporated gamification [30].

A need or desire for more sessions was reported by some participants [29,42], and there was some variation around how difficult participants thought the content of the interventions should be [42]. However, where it was provided, a personalized approach was appreciated by participants [29]. Overall, positive perceptions of the interventions, regardless of how they were delivered (at home, online, or in groups), were reported by participants in all 9 studies. This included some subpopulations such as those living in rural areas [40,47], being at risk of social isolation [28,29], or being homebound [42].

### Cost-Effectiveness of Interventions

There was limited evidence on the cost-effectiveness of interventions, with only 1 study reporting this outcome. The cost-effectiveness of a community-based information and communication technologies training intervention for older adults with visual impairment was assessed by 1 study [45]. The results found that the intervention was cost-effective but only under the assumption that the effects of the training remain consistent for 10 years. When the willingness-to-pay threshold was €20,000 per year of well-being (a currency exchange rate of €1= US $1.18 was applicable), the probability that the training would be cost-effective was 91%, when including only the costs of the training.

## Discussion

### Main Findings

The effectiveness of interventions that aimed to increase a range of digital skills and technology use in older adults in the social care domain was assessed in this rapid review. Insights into how interventions could improve digital literacy, digital use, perceptions of technology, and overall acceptability among older adults were identified. Positive results were consistently observed across different subpopulations, including those living in rural areas, socially isolated individuals, homebound older adults, those with visual impairments, and those belonging to lower socioeconomic groups. The improvements seen were noted in both community and home-based settings. However, as the evidence base was limited by low-quality research, further high-quality research is needed to draw firm conclusions.

### Comparison With Existing Literature

The evidence in support of intervention effectiveness identified in this rapid review is promising, as both older age and deprivation are associated with an increased likelihood of digital exclusion [48,49]. Digital exclusion among older adults varies widely across countries. A recent study using data from 5 international ageing cohorts found the highest exclusion rates in Romania (939/1582, 59%), Bulgaria (689/1012, 68%), and India (38,321/42,083, 91%), driven by socioeconomic and health- and aging-related factors [50]. The authors call for inclusive digital strategies tailored to older populations, especially in resource-limited settings. In the European Union, 43% of older adults aged 65-74 years had not used the internet in 2019, and only 16% had above-basic digital skills [51]. Together, these findings highlight the global challenge of digital exclusion for older adults and demonstrate the need for tailored interventions. Valuable insights into the effectiveness, acceptability, and preferences of older adults undertaking digital training were identified in this rapid review. To maximize effectiveness, those designing interventions to address digital exclusion in older adults may benefit from considering the preferences or specific needs of older learners. Similar findings were reported in a recent systematic review; intergenerational learning, game-based, and peer learning interventions were found to effectively enhance the cognitive, social, attitudinal, and health-related needs of older adults, and future interventions that are personalized and remove any practical barriers were suggested by the study authors [52]. It has been suggested in another systematic review that effective strategies for enhancing the digital skills of older adults should focus on designing training based on the needs and preferences of the participants [53]. Furthermore, consistent with the findings of this rapid review, collaborative learning in informal learning settings was identified as 2 key themes in digital training for older adults [53]. A range of preferences of older adults in relation to learning new digital skills has been highlighted by the UK Consumer Index, including whether the learning was “live” or self-directed, was taught by professionals or family and friends, and whether learning was provided face-to-face or through recorded tutorials [9]. The findings from the UK Consumer Index are supported by the findings from this rapid review, as, despite their varying approaches and delivery modes, the interventions were found to be acceptable by the participants. The rapid review also identified information and communication technologies training as cost-effective, although this evidence was limited to a single study involving older adults with visual impairment [45].

The identified interventions also appear effective in promoting digital use among older adults, with particularly positive outcomes among rural and homebound populations. These populations are pertinent given that, as well as older age, rurality [54] and disability [55] both increase the likelihood of digital exclusion. Improved perceptions of technology and the ability to engage with the digital world were consistently reported at the end of interventions. This finding is crucial for overcoming barriers such as fear and anxiety, which are well-documented obstacles to digital inclusion [13,15,17] and contribute to digital exclusion among the older population. Improvements in perceived abilities were consistent regardless of the intervention delivery format or setting across studies.

Positive perceptions of the interventions were highlighted through participant feedback. High acceptance was reported across different countries, population groups, and intervention delivery methods. Although some suggestions for improving the interventions were noted, participants largely valued and benefited from the training provided. Additionally, the provision of digital devices and internet access as part of the intervention was assessed by a small subset of studies demonstrating that these approaches could enhance online engagement by addressing affordability and accessibility challenges. However, participants emphasized the need for continued support after the intervention to maintain sustained digital engagement.

### Strengths and Limitations of the Available Evidence

A range of interventions were assessed using differing delivery methods and included a range of subpopulation groups, providing a wide-ranging assessment of interventions to reduce digital exclusion. In addition, given the fast pace of technology development, the included studies were limited to those published since 2018 to ensure the recency and relevance of internationally published evidence available to inform policy and practice.

Several evidence gaps were identified in the evidence base, notably an absence of recent studies conducted within the United Kingdom. There was also a lack of research focused on interventions to improve access to, or affordability of, the internet and digital technologies to overcome digital exclusion. In addition, there appears to be a paucity of cost-effectiveness analyses. Cost-effectiveness was assessed by only 1 study identified in this rapid review, which investigated an information and communication technologies training intervention in the Netherlands [45]. Despite reporting positive outcomes, more research is needed to comprehensively assess the cost-effectiveness of such interventions. Due to the paucity of UK-based research on this topic area, we cannot be certain that the interventions, irrespective of location or delivery method, would apply to the UK context. Furthermore, no studies specifically addressed language barriers, for example, that may be experienced by people whose first language is Welsh, revealing a further evidence gap.

While 3 of the included studies were randomized controlled trials and were determined to be of moderate quality, overall, the evidence base was limited by low-quality research with a high risk of bias, highlighting a paucity of studies using robust experimental methods to determine the effectiveness of interventions addressing digital exclusion in older adults. Of the 10 nonrandomized controlled trials, all were determined to be of low quality, and few undertook between-group comparisons and only investigated within-group differences. The 8 uncontrolled before-and-after studies were also all determined to be of low quality and were limited by a lack of an external control group. In addition, only a small number of studies performed longer-term follow-up, so the sustainability of the intervention effectiveness over time is largely unknown. Participant outcomes were also commonly self-reported and were not obtained using objective measures, limiting our ability to make firm inferences relating to intervention effectiveness. Given the quality of the evidence base, it is not possible to draw definitive conclusions regarding the effectiveness of the interventions included in this rapid review. As such, the overall certainty in the findings is low, and the conclusions should be interpreted with caution.

### Strengths and Limitations of This Rapid Review

An important strength is that studies included in this rapid review were systematically identified through a comprehensive search of electronic databases, as well as using supplementary search methods. However, it is possible that additional relevant publications published before 2018 have been missed. The broad definition of digital literacy used to inform eligibility is also a strength, as it enabled the identification and inclusion of a wide range of research addressing various facets of digital literacy. However, this broad approach made it challenging to synthesize the findings across studies due to the varied terminology, definitions, and measurements used to assess the different outcomes. Every effort was made to conduct a robust synthesis of study findings; however, the poor reporting of intervention methods and results in several studies made interpretations challenging. Another strength is that each stage of the rapid review was consistency checked for accuracy, and any issues that arose were resolved through discussion within the team.

### Implications for Policy and Practice

Overall, the evidence identified suggests that a range of interventions can increase digital skills and technology use in older adults, which in turn may reduce digital exclusion. The findings of this rapid review can inform the development and delivery of future interventions. However, it is important to consider the context in which the included interventions were used and the lack of certainty of the findings, given the methodological limitations identified.

Lower income and socioeconomic status, as well as costs of acquiring and using technology, are established barriers to digital inclusion [14,56]. To achieve improved and sustained digital inclusion in older adults, evidence suggests it may be important to ensure structural barriers, such as access to the internet and affordability of devices, are removed. However, it is unclear what the cost implications may be to deliver this, or if these barriers could be reduced by raising awareness of social tariffs available in the United Kingdom for those receiving pension credit [57]. In practice, providing education and training to older adults without considering these barriers may exacerbate existing inequalities. It is also important to consider that digital devices and services are continually evolving, meaning education and training provision may need to be ongoing, or regularly updated to ensure sustained digital literacy and technology use.

Although it was reported by study participants that they generally appreciated and benefitted from the interventions, it is important to consider that older adults retain the right to choose whether or not to interact with essential services physically (offline) or digitally. While several strategies and frameworks emphasize the importance and need to equip individuals of all ages with the motivation, access, skills, or confidence to engage with digital technologies [58,59], services undergoing digitization may need to find ways to encourage and support older adults to engage as they wish to do so. Therefore, alternative methods of accessing these services should remain available where possible, or it may exacerbate exclusion by leaving some people behind. There are several barriers to engaging with the digital world that are particularly pertinent within the older adult population, including fear, anxiety, stereotypes, and stigmas, as well as costs or disabilities [13,15,17,55], which need to be addressed in order to reduce digital exclusion. It is also important to consider that the digital skills and physical ability of the older adult population are likely to vary greatly. For example, working older adults who use digital technologies regularly may have different needs to reduce their digital exclusion compared to those who have been retired for many years or those who do not regularly interact with digital technologies.

Practitioners working to address digital exclusion should be aware of the wide-ranging factors that may contribute to digital exclusion as well as the varying skills, abilities, and needs of older adults themselves. This includes structural barriers, such as access to the internet and affordability of devices, and perceptual barriers such as a lack of confidence, fear, and anxiety, or perceived lack of abilities.

### Implications for Future Research

The findings of this rapid review highlight a paucity of randomized controlled trials, suggesting there is a need for high-quality research with robust evaluation to advance the topic area. It may also be beneficial for researchers to adopt standardized definitions and measurement tools, promoting greater consistency and comparability in study findings in relation to digital exclusion to assist those considering implementation.

Future research should consider how older adults want to access digital services and the diverse range of digital skills required by older people to reduce digital exclusion, such as learning how to use email, navigating the internet, and the ability to identify risks, such as phishing emails, as these will provide important insights when creating such interventions.

The findings of this rapid review identified a range of interventions focused on subpopulations of older adults, such as those from small or rural towns, low socioeconomic status, at risk of social isolation, homebound, or with visual impairment. However, further research is needed to strengthen the evidence base of specific groups, particularly those at greater risk of digital exclusion, such as those with disabilities and those who live in rural areas [54,55]. This is important to better understand the specific needs of these population groups, so interventions can be tailored to their individual requirements. Furthermore, there is a need for research focused on overcoming language barriers when addressing digital exclusion in older adults; a key challenge highlighted by stakeholders in our Welsh context [60] and not identified in the evidence base. Lastly, further evidence is needed to determine if digital exclusion can be addressed in a cost-effective way and to determine potential cost savings in both the older adult population more generally, as well as the subpopulations within it.

Overall, priorities for future research should include identifying the varying needs of specific subpopulations within older adults and tailoring interventions to address these needs. Future research should also assess interventions to address other factors associated with digital exclusion, such as improving access to, or affordability of, the internet and digital technologies.

### Conclusions

The potential benefits of interventions aimed at improving a range of digital skills and increasing technology use in older adults, which could help to address digital exclusion, were highlighted by this rapid review. The evidence suggests that older adults are accepting of these interventions and empowered by them; however, consideration should be given to those who do not wish to engage with the online world to ensure they are not left behind. The lack of high-quality research identified in this rapid review limits the overall confidence in the findings and suggests that further research is needed with robust evaluations to assess how digital exclusion can be addressed in older adults. Further research should focus on older adults most likely to experience digital exclusions, such as those with disabilities or those who live in rural areas, as they may have differing needs from the wider older adult population. Considerations around how older adults want to engage with digital technologies can be used to tailor interventions to meet these needs. Future research should also assess interventions to address other factors associated with digital exclusion, such as improving access to, or affordability of, the internet and digital technologies for older adults.

## Appendix

Multimedia Appendix 1 Search strategies.

Multimedia Appendix 2 Quality appraisal results.

Multimedia Appendix 3 Summary of interventions.

Multimedia Appendix 4 Data extraction of included studies.

Multimedia Appendix 5 PRISMA Checklist.

## Abbreviations

PRISMA: Preferred Reporting Items for Systematic Reviews and Meta-Analyses
