# Supplementary material for: Effectiveness of Interventions for Addressing Digital Exclusion in Older Adults in the Social Care Domain: Rapid Review
Source: JMIR Aging. 2025 Dec 30;8:e70377. doi: 10.2196/70377 (PMC12826648; doi:10.2196/70377)
Supplement: Multimedia Appendix 1 [file aging_v8i1e70377_app1.docx]

**Search strategy used for Social Policy and Practice (Ovid)**

1. ("Digital exclusion" or "Digital inclusion" or "Digital divide*" or "Digital division*" or "Digital disparit*" or "Digital gap*" or "Digital inequalit*" or "digital training" or "Technostress" or "Digital literacy" or "Digital skills" or "Computer literacy" or "ICT literacy" or "computer skills" or "ICT skills" or "information and communicat* technolog*").ti,ab. 1696

2. (elder* or old* or ageing or aging or senior or seniors or carer* or geriatric* or Centarian* or centenarian* or eldest or frail* or geriatri* or nonagenarian* or octagenarian* or octogenarian* or "old age*" or "older adult*" or "older age*" or "older patient*" or "older people" or "older person*" or "older population" or "older subject*" or oldest or senium or septuagenarian* or supercentenarian* or "older female*" or "older male*" or "older man" or "older men" or "older woman" or "older women").ti,ab. 95831

3. 1 and 2 486

4. limit 3 to yr="2018 - 2024" 165

**Search strategy used for Scopus (Elsevier)**

TITLE-ABS("Digital exclusion" or "Digital inclusion" or "Digital divide*" or "Digital division*" or "Digital disparit*" or "Digital gap*" or "Digital inequalit*" or "digital training" or "Technostress" or "Digital literacy" or "Digital skills" or "Computer literacy" or "ICT literacy" or "computer skills" or "ICT skills" or "information and communicat* technolog*") AND TITLE-ABS(intervention* or program* or training or strategy* or approach* or evaluat* or solution* or packag* or mentor*) AND TITLE-ABS(elder* or ageing or aging or senior or seniors or carer* or geriatric* or Centarian* or centenarian* or eldest or frail* or geriatri* or nonagenarian* or octagenarian* or octogenarian* or "old age*" or "older adult*" or "older age*" or "older patient*" or "older people" or "older person*" or "older population" or "older subject*" or "older female*" or "older male*" or "older man" or "older men" or "older woman" or "older women" or oldest or senium or septuagenarian* or supercentenarian*) AND PUBYEAR > 2017 AND PUBYEAR < 2025 AND ( EXCLUDE ( DOCTYPE,"re" ) OR EXCLUDE ( DOCTYPE,"cp" ) OR EXCLUDE ( DOCTYPE,"ch" ) OR EXCLUDE ( DOCTYPE,"cr" ) OR EXCLUDE ( DOCTYPE,"bk" ) OR EXCLUDE ( DOCTYPE,"no" ) OR EXCLUDE ( DOCTYPE,"ed" ) OR EXCLUDE ( DOCTYPE,"le" ) ) AND ( LIMIT-TO ( LANGUAGE,"English" ) )  - 964

**Search strategy used for Sociology collection (ProQuest)**

(ti,ab("Digital exclusion" OR "Digital inclusion" OR "Digital divide*" OR "Digital division*" OR "Digital disparit*" OR "Digital gap*" OR "Digital inequalit*" OR "digital training" OR "Technostress" OR "Digital literacy" OR "Digital skills" OR "Computer literacy" OR "ICT literacy" OR "computer skills" OR "ICT skills" OR "information and communicat* technolog*") AND ti,ab(elder* OR old* OR ageing OR aging OR senior OR seniors OR carer* OR geriatric* OR Centarian* OR centenarian* OR eldest OR frail* OR geriatri* OR nonagenarian* OR octagenarian* OR octogenarian* OR ("old age" OR "old aged" OR "old agency" OR "old ages") OR ("older adult" OR "older adults") OR ("older age" OR "older aged" OR "older ages") OR ("older patient" OR "older patients") OR "older people" OR ("older person" OR "older persons") OR "older population" OR ("older subjects") OR oldest OR senium OR septuagenarian* OR supercentenarian* OR ("older female" OR "older females") OR ("older male" OR "older males") OR "older man" OR "older men" OR "older woman" OR "older women")) AND pd(20180101-20231231) - 366
