# Supplementary material for: Effectiveness of Interventions for Addressing Digital Exclusion in Older Adults in the Social Care Domain: Rapid Review
Source: JMIR Aging. 2025 Dec 30;8:e70377. doi: 10.2196/70377 (PMC12826648; doi:10.2196/70377)
Supplement: Multimedia Appendix 2 [file aging_v8i1e70377_app2.docx]

**Quality appraisal results for quasi-experimental studies**

| **Study** | **JBI Appraisal Items – Quasi-experimental studies** | | | | | | | | | **Overall quality** |
| --- | --- | --- | --- | --- | --- | --- | --- | --- | --- | --- |
|  | **Q1.** | **Q2.** | **Q3.** | **Q4.** | **Q5.** | **Q6.** | **Q7.** | **Q8.** | **Q9.** |  |
| Castilla et al. (2018) | Y | Y | Y | N | N | N/A | Y | U | Y | **Low** |
| Choi and Park (2022) | Y | U | Y | Y | N | Y | Y | U | Y | **Low** |
| Elbaz et al. (2023) | Y | Y | Y | N | N | N/A | Y | U | Y | **Low** |
| Gadbois et al. (2022) | Y | Y | Y | N | N | N/A | Y | U | Y | **Low** |
| Garcia et al. (2022) | Y | U | Y | Y | N | N | Y | U | Y | **Low** |
| Holguin-Alvarez et al. (2020) | Y | Y | Y | Y | N | N | Y | U | U | **Low** |
| Lee and Kim (2019) | Y | Y | Y | N | N | N/A | Y | Y | Y | **Low** |
| Lee et al. (2022a) | Y | N | Y | Y | N | N | Y | U | Y | **Low** |
| Lee et al. (2022b) | Y | N | Y | Y | N | N | Y | Y | Y | **Low** |
| Ma et al. (2020) | Y | Y | Y | N | N | N/A | Y | Y | Y | **Low** |
| Martínez-Alcalá et al. (2018) | Y | Y | Y | Y | N | N | Y | U | Y | **Low** |
| Martínez-Alcalá et al. (2021) | Y | U | Y | N | Y | N | Y | U | Y | **Low** |
| McCosker et al. (2020) | Y | Y | Y | N | N | N/A | Y | U | Y | **Low** |
| Moore & Hancock (2022) | Y | U | Y | Y | N | Y | Y | U | Y | **Low** |
| Ngiam et al. (2022) | Y | Y | Y | Y | N | Y | Y | U | Y | **Low** |
| Patty et al. (2018) | Y | Y | Y | N | Y | N/A | Y | U | Y | **Low** |
| Quialheiro et al. (2023) | Y | Y | Y | N | Y | N/A | Y | Y | Y | **Low** |
| Seaton et al. (2023) | Y | Y | Y | N | N | N/A | Y | Y | Y | **Low** |
| Key: Y=Yes, N=No, U=unclear, N/A=not applicable | | | | | | | | | | |

1. Is it clear what is the cause and what is the effect?

2. Were the participants included in any comparisons similar?

3. Were the participants included in any comparisons receiving similar treatment/care, other than the exposure or intervention of interest?

4. Was there a control group?

5. Were there multiple measurements of the outcome both pre and post the intervention/ exposure?

6. Was follow up complete and if not, were differences between groups in terms of their follow up adequately described and analysed?

7. Were the outcomes of participants included in any comparisons measured in the same way?

8. Were outcomes measured in a reliable way?

9. Was appropriate statistical analysis used?

**Quality appraisal results for randomised controlled trials**

| **Study** | **JBI Appraisal Items – Randomised Controlled Trial** | | | | | | | | | | | | | **Overall quality** |
| --- | --- | --- | --- | --- | --- | --- | --- | --- | --- | --- | --- | --- | --- | --- |
|  | **Q1** | **Q2** | **Q3** | **Q4** | **Q5** | **Q6** | **Q7** | **Q8** | **Q9** | **Q10** | **Q11** | **Q12** | **Q13** |  |
| Arthanat (2021) | Y | U | Y | U | U | U | Y | N | Y | Y | U | Y | Y | **Moderate** |
| Czaja et al. (2018) | U | U | Y | U | U | Y | Y | N | Y | Y | U | Y | Y | **Moderate** |
| Fields et al. (2021) | U | U | Y | U | U | U | Y | Y | U | Y | U | Y | Y | **Moderate** |
| Key: Y=Yes, N=No, U=unclear, N/A=not applicable | | | | | | | | | | | | | | |

Q1. Was true randomisation used for assignment of participants to treatment groups?

Q2. Was allocation to treatment groups concealed?

Q3. Were treatment groups similar at the baseline?

Q4. Were participants blind to treatment assignment?

Q5. Were those delivering treatment blind to treatment assignment?

Q6. Were outcomes assessors blind to treatment assignment?

Q7. Were treatment groups treated identically other than the intervention of interest?

Q8. Was follow up complete and if not, were differences between groups in terms of their follow up adequately described and analysed?

Q9. Were participants analysed in the groups to which they were randomised?

Q10. Were outcomes measured in the same way for treatment groups?

Q11. Were outcomes measured in a reliable way?

Q12. Was appropriate statistical analysis used?

Q13. Was the trial design appropriate, and any deviations from the standard RCT design (individual randomisation, parallel groups) accounted for in the conduct and analysis of the trial?
