# Supplementary material for: Effectiveness of Interventions for Addressing Digital Exclusion in Older Adults in the Social Care Domain: Rapid Review
Source: JMIR Aging. 2025 Dec 30;8:e70377. doi: 10.2196/70377 (PMC12826648; doi:10.2196/70377)
Supplement: Multimedia Appendix 3 [file aging_v8i1e70377_app3.docx]

| **Citation (Country)** | **Name and type of intervention/control** | **Intervention characteristics** |
| --- | --- | --- |
| **Arthanat (2021)**  **(USA)** | **Intervention:** The i-CHATT program**.** An individualised inter-generational ICT training programme to facilitate ICT use in older adults from rural areas.  **Control:** No ICT intervention. | **Setting:** Home-based  **Mode of delivery:** Three home visits each lasting about 90 minutes to two hours    **Duration/intensity:** 3 months  **Intervention details:**   - Participants received hands-on one-to-one support from a coach (an undergraduate student) - An ICT priority checklist was given to participants to choose their priorities and set goals for the programme. This was used by coaches to inform sessions and track progress throughout the programme - A community Facebook group with trainees and coaches was created to promote peer-to-peer learning and share achievements - Participants were loaned an iPad for the course and the duration of follow-up - The intervention formed part of an occupational therapy programme - Data were collected at baseline and in six-month intervals for 24 months |
| **Castilla et al. (2018)**  **(Spain)** | **Intervention:** Coaching older adults in rural areas to use a social network (Butler 2.0) consisting of multiple applications to improve digital literacy and increase digital inclusion.  **Comparator:** No control, comparisons with baseline scores. | **Setting:** Elderly Leisure Centre  **Mode of delivery:** 8 sessions delivered weekly in groups of up to 6 people at an elderly leisure centre.  **Duration/intensity:** 8 weeks.  **Intervention details:**   - Session 1 to 5 covered the basics aspects of the Butler 2.0 website. This included, user registration, creating a profile and information about each resource. - Session 6 to 8 included internet searching and independent exploration of the system’s resources - After completing the eight sessions, all the participants were invited to a focus group - The intervention used accessible software and hardware to assist elderly participants - Butler 2.0 is a system aimed at improving the social support network and quality of life of the elderly population through the use of new technologies that help to remove the digital barrier for this sector of the population, encouraging their e-inclusion. - Data were collected 1) before the use of the system; 2) after the first contact with the system (post session 1); 3) after session 5; and 4) after the last session of use |
| **Choi & Park (2022)**  **(South Korea)** | **Intervention:** Educational decision tree and game to improve IT knowledge**.**  **Comparator:** General Internet and digital device use education. | **Setting:** Education centre  **Mode of delivery:** 10 sessions delivered in groups  **Duration/intensity:** Not stated.  **Intervention details:**   - The intervention combines a decision tree with a game and allows the elderly to acquire IT knowledge while playing a game naturally. - The educational programme was largely divided into 'Understanding the decision tree' and 'Applying the decision tree.' - The educational game was called 'Save the Titanic using the decision tree', researchers introduced a Titanic movie, historical events related to the Titanic, and the causes of the sinking. By incorporating a humanities approach into IT education, it was possible to increase the understanding of unfamiliar IT and to feel how to apply IT to real-life problem-solving. - The researchers designed the education program for the elderly in their 60s or older, and it can be used at lifelong education centres or universities for the elderly. - Data were collected pre- and post-intervention |
| **Czaja et al. (2018)**  **(USA)** | **Intervention:** A specially designed Personal Reminder Information and Social Management (PRISM) system for older adults at risk of social isolation, with training and instructional support. PRISM included features that provided easy access to resources and information sources, and opportunities for engagement and communication.  **Comparator:** Provision of a notebook that contained paper content similar to that contained in the intervention. | **Setting:** Home-based  **Mode of delivery:** 4 initial home sessions, telephone check-ins, and home and telephone follow-ups.  **Duration/intensity:** 12 months.  **Intervention details:**   - PRISM included: Internet access (with vetted links to sites such as NIHSeniorHealth.Gov), an annotated resource guide, a dynamic classroom feature, a calendar, a photo feature, E-mail, games, and online help - Participants in the intervention group were provided with a PC, keyboard, mouse, printer, and internet access and were compensated $25 per assessment. They were allowed to keep the hardware after completing the programme - Participants were given the opportunity to be listed as a “PRISM Buddy” that would allow them to have contact with people who had similar interests - Data were collected at baseline and at 6 and 12 months post-randomisation |
| **Elbaz et al. (2023)**  **(Canada)** | **Intervention:** A brief online digital literacy intervention (pilot study) covering a range of skill-based topics including safety and security.  **Comparator:** No control, comparisons with baseline measures. | **Setting:** Online (During the COVID-19 pandemic)  **Mode of delivery:** 8 × 1.5 h group intervention sessions delivered weekly by a trainer and two co-facilitators remotely via Zoom. In-person meetings on a request basis.  **Duration/intensity:** 4 weeks.  **Intervention details:**   - Sessions aimed to equip participants with basic digital literacy skills. The topics of sessions targeted their ability to use email, Zoom, tablet and smartphone, Facebook and WhatsApp, browsing online, shopping online and browsing for entertainment - Data were collected pre- and post-intervention |
| **Fields et al. (2021)**  **(USA)** | **Intervention:** Tech Allies. One-to-one digital training sessions for isolated older adults.  **Control:** A 2-month waitlist group. | **Setting:** Home-based  **Mode of delivery:** 8, one-to-one sessions delivered weekly by volunteers  **Duration/intensity:** 2 months.  **Intervention details:**   - Tech Allies was a partnership among Little Brothers - Friends of the Elderly (LBFE), a volunteer-based organisation that provides home visits for isolated and lonely older adults; Community Tech Network (CTN), a digital literacy training organisation - Participants were given a tablet and broadband access which they could keep after completing the programme. - CTN created a learner booklet for each participant, outlining curriculum topics by week, including step-by-step visual guides and practice exercises. The eight sessions covered (a) getting to know the iPad (hardware, touch screen, typing, voice dictation); (b) using the iPad (operating system, getting online, searching for information); (c) online safety (passwords, phishing, viruses); (d) email (creating an account, sending email, using the camera); (e) email safety (opening and replying to emails, identifying spam); (f) communicating via apps and FaceTime; (g) online communities (social media); and (h) having fun and wrap-up (entertainment, learning, shopping) - Data were collected pre- and post-intervention |
| **Gadbois et al. (2022)**  **(USA)** | **Intervention:** Talking Tech (pilot study). Technology training and assistance to promote digital literacy, with the primary aim of reduce loneliness and social isolation in homebound older adults. Participants received one-to-one support and were provided with self-directed learning materials.  **Comparator:** No control, comparisons with baseline measures. | **Setting:** Home-based  **Mode of delivery:** One-to-one in-home training for the first 4 weeks, scheduled telephone support for the following 8 weeks, for the final 2 weeks of the programme support was available if the participant reached out by phone with questions  **Duration/intensity:** 14 weeks.  **Intervention details:**   - The Talking Tech intervention was embedded within and delivered by a home-delivered meals program, and provided training and assistance to participants using trained volunteers, called TechMates - Participants were paired with a TechMate and given a tablet and a hotspot connection for 1 year (if they did not already have in-home internet access). While the intervention lasted 14 weeks, participants were encouraged to keep their tablets and maintain internet connection permanently - Training and support focused on how to use a tablet, access the internet, and participate in a virtual senior centre program, called Well Connected. Well Connected is a national phone- and internet-based program, which provides over 70 sessions per week on topics including virtual travel, support and conversation groups, bingo, and language learning, among others. - Relationships between participants and TechMates and between participants and the home-delivered meal provider staff were not severed upon completion of the 14 weeks, in case of participant questions or challenges - Data were collected pre- and post-intervention (week 15) |
| **Garcia et al. (2022)**  **(Latvia, Poland, Portugal and the UK)** | **Intervention:** Erasmus+ project ICTskills4All. Different educational delivery approaches to improve digital skills, 1) intergenerational approach, 2) peer-to-peer approach, 3) online learning  **Comparator:** Three different methods of delivery | **Setting:** Classroom based (intergenerational and peer-to-peer groups) and virtual (online group)  **Mode of delivery:** 8, 2-hour sessions in groups of 12 (intergenerational), 18 (peer-to-peer), and 9 (online).  **Duration/intensity:** Not stated.  **Intervention details:**   - Main objectives of all interventions: preparation and motivation for self-continued training, using e-learning + support overcoming the fear of technology and gaining skills to independently use a computer strengthening trust in success - The learning assistants could be young people between 12 and 26 years old (intergenerational approach) or adults over 55 years old (peer-to-peer). The online approach occurred due to the COVID-19 pandemic, which prevented face-to-face meetings during 2020. - Data were collected pre- and post-intervention |
| **Holguin-Alvarez et al. (2020)**  **(Peru)** | **Intervention:** Social media programme designed to increase digital skills in communities in vulnerable contexts (Low SES). The educational activities were based on the use of Facebook, WhatsApp, Twitter and Gmail.  **Control:** No intervention. | **Setting:** Elderly centre  **Mode of delivery:** 50 sessions lasting 30 minutes each  **Duration/intensity:** Not stated.  **Intervention details:**   - The programme used a graded approach which was divided into 4 phases: 1) a familiarisation phase, 2) introduction activities, 3) network registration, and 4) digital interactivity - Data were collected pre- and post-intervention |
| **Lee & Kim (2019)**  **(USA)** | **Intervention:** Intergenerational Mentor-Up (IMU). Guided learning opportunities for undergraduate students to teach older adults from low income areas IT skills and reduce their social isolation.  **Comparator:** No control, comparisons with baseline measures. | **Setting:** Senior centres or housing facilities  **Mode of delivery:** 6 sessions in groups of 6-8  **Duration/intensity:** Not stated.  **Intervention details:**   - In each class, youth mentors and senior mentees sat around long tables or in a big circle during the initial part of class before breaking into smaller groups to work on individualised tasks. Seniors were asked to state their learning goals for the sessions. The subsequent discussion centered on what the older adults wanted to learn about technology, and the ways in which they currently use technology. Each youth was paired with a senior mentee to help them with their particular issues - Youth mentors were students. The programme gave opportunity to refine their interpersonal skills and receiving community service learning credits in return - Data were collected pre- and post-intervention |
| **Lee et al. (2022a)**  **(South Korea)** | **Intervention:** Social prescribing through digital literacy education for older adults in Wonju-si. A digital literacy education programme to improve smartphone usage competency in older adults from rural areas.  **Control:** No intervention. | **Setting:** 5 locations including Y University Healthy City Research Center, the Wonju Senior Center and small libraries.  **Mode of delivery:** One 60 minute session per week  **Duration/intensity:** 6 weeks.  **Intervention details:**   - Education topics included: Basic smartphone operation, sending text messages, taking and sharing photos, using social apps, search engine applications and internet banking - Data were collected pre- and post-intervention |
| **Lee et al. (2022b)**  **(South Korea)** | **Intervention:** The Intergenerational Forum (IF). Class educational programme providing guided instruction and intergenerational exchange between youths (mentors) and older adults (mentees).  **Control:** No intervention. | **Setting:** 2 large elderly centres  **Mode of delivery:** Six cohort of classes of 8-10  **Duration/intensity:** 12 weeks.  **Intervention details:**   - The programme was based on collaborative learning theory, each IF class is structured where by older mentees come together to complete their learning goals. - During the first half of class, basic instruction was given to provide older mentees the opportunity to learn about the Internet and their own unique computerised devices. Based on skills and interests, each youth mentor was paired with an older mentee to assist with their particular issues. The second half of classes focused on participants’ individualised digital technology needs and interests - Data were collected pre- and post-intervention |
| **Ma et al. (2020)**  **(China)** | **Intervention:** A video tutorial-based intervention which aimed to enhance technology acceptance in older adults. The video tutorials provided demonstrations of how to complete basic tasks using a tablet.  **Comparator:** Three intervention groups that differed according to the model in the videos (a child model, young adult model, or older adult model). | **Setting:** Senior citizen centre  **Mode of delivery:** Each intervention delivery method consisted of 3 short sessions conducted on the same day  **Duration/intensity:** Not stated.  **Intervention details:**   - The videos included a simple introduction of a tablet’s physical appearance, buttons, hand gestures, and scenario demonstrations by the three models. There were four scenarios simulated in the video: (1) route-planning using the transportation information app, (2) playing a movie with the video player on the tablet and enjoying it, (3) having a conversation with family members using with communication app, and (4) heart rate measurement using the health monitoring app. After demonstrations, participants were given time to practice what they had been shown. - Other than different role models, each participant in the three groups received the same training content and procedure. Models in three generations were recruited for video recording, including a child aged 10, a young adult aged 23, and an older adult aged 68 - Video clips were no longer than 15 minutes - Data were collected pre- and post-intervention |
| **Martínez-Alcalá et al. (2018)**  **(Mexico)** | **Intervention:** A digital literacy workshop for older adults delivered either face-to-face by a tutor, blended workshops based on a learning management system  **Comparator:** Comparisons within and between the face-to-face and blended delivery models. | **Setting:** Face-to-face workshop: classrooms and blended workshops used a learning management system that could be accessed via mobile device from anywhere at any time.  **Mode of delivery:** Face-to-face workshop: 3 face-to-face sessions delivered by a tutor to groups of 15-25 in computer rooms with internet connection. The blended intervention used a learning management system for participants to work through.  **Duration/intensity:** 4 months.  **Intervention details:**   - Both intervention groups content consisted of three lessons namely, Introduction to ICT, Computer Programs and Getting to Know the Internet, with a total of 16 topics. - Face-to-face workshop: Regarding learning methods, each student had a printed manual with the topics that would be studied in the workshop. For the instruction of digital skills, the tutor used digital presentations and a projector as support material. At the beginning of each class, the tutor asked random questions to each student so that they could remember the concepts and topics seen in the previous classes. - Blended Workshops Based on a Learning Management System: regarding learning methods, the lesson sequence was organised in initiation, development and closure activities. - Data were collected pre- and post-intervention |
| **Martínez-Alcalá et al. (2021)**  **(Mexico)** | **Intervention:** Digital literacy programme delivered either as a blended, transition (part digital), and fully digital format. The workshops consisted of five levels of digital literacy which have been designed according to the needs and characteristics of the elderly, so that they can increasingly acquire more sophisticated digital skills.  **Comparator:** Comparisons within and between the different delivery models. | **Setting:** Blended learning: community classroom setting, Transition treatment and Digital treatment: remote  **Mode of delivery:** Blended participants interacted face-to-face and accessed the digital literacy course online through a learning management system. Participants attended the computer classrooms twice a week for 2-hours. Transition participants took classes as per the blended protocol, but this stopped due to COVID-19 restrictions, so were introduced to remote learning modalities, using Zoom for the delivery of classes. Digital participants attended a completely online modality.  **Duration/intensity:** 3-4 months.  **Intervention details:**   - The intervention changed from blended delivery to digital delivery in response to the COVID-19 pandemic - The five levels of workshop were Basic 1, Basic 2, Intermediate, Upper Intermediate, and Advanced. Participants were able to progress through the levels - Personalised advice and telephone assistance was offered to participants enrolling onto the workshops in the digital treatment so that they could set up and use the device to complete the course - Personal counselling was offered across all delivery methods. This was in-person during the blended learning phase and via WhatsApp during the digital treatment - Data were collected pre- and post-intervention |
| **McCosker et al. (2020)**  **(Australia)** | **Intervention:** Be Connected. A national digital inclusion programme to provide older learners with personalised, face-to-face and self-paced learning across Australia.  **Comparator:** No control, comparisons with baseline measures. | **Setting:** Face-to-face sessions were offered by community-based organisations as well as online learning through a government Web portal  **Mode of delivery:** 12 online learning modules with face-to-face support from a network of community-based organisations.  **Duration/intensity:** Not stated.  **Intervention details:**   - The two core components to the programme’s design were: online learning modules presented through a government Web portal, and face-to-face support provided by a network of community-based organisations - A national network of community organisations (Network Partners) offering personalised and face-to-face support for older learners, coordinated and supported by the DSS funded National Network Manager, Good Things Foundation (GTF). GTF has provided Network Partners with professional development, up-skilling, one-on-one coaching - Modules cover a variety of topics covering, for example, device and operating systems, online safety, email, social media, and some interest areas such as online videos and genealogy - Sessions were targeted for very basic users as well as more experienced users - Data were collected after one month of registration to the portal, and then again four months after that |
| **Moore & Hancock (2022)**  **(USA)** | **Intervention:** The MediaWise for Seniors course. A self-directed online course which taught digital media literacy skills and techniques helpful for verifying the credibility of information online, to improve resilience to fake news.  **Control:** no intervention. | **Setting:** The course was completed virtually  **Mode of delivery:** A self-directed online course which took approximately 1-hour to complete at participant’s own pace.  **Duration/intensity:** Participant’s completed the course at their own pace, no set timeframe stated.  **Intervention details:**   - The contents of the course were highly multimodal. Information was presented as text, in photos and infographics, through instructional videos, and through interactive examples where participants were walked through examples of encountering online misinformation. - Data were collected pre- and post-intervention |
| **Ngiam et al. (2022)**  **(Singapore)** | **Intervention:** Project Wire Up. A volunteer-led, one-on-one, and home-based digital literacy programme for older adults in vulnerable contexts (Low SES)  **Control:** Older adults on a waitlist (no intervention). | **Setting:** Home-based, one-to-one support  **Mode of delivery:** 6, 1-2-hour sessions  **Duration/intensity:** Typically 3 months  **Intervention details:**   - Digital skills training was conducted during the home visits by trained volunteers, who guided older adults through a tiered curriculum of increasing difficulty that could be tailored to the needs of older adults. - Older adults were equipped with smartphones and internet connection - At the base level, older adults were taught the basic use of the phone, such as making calls and sending messages, before progressing to other social telecommunication platforms (e.g. WhatsApp) or entertainment platforms (e.g. YouTube). More digitally savvy older adults were taught advanced smartphone functions such as accessing government websites, making purchases, or paying bills on the web. At the end of the program, older adults would be connected to existing formal and informal networks through platforms such as mobile communication apps. - Data were collected pre- and post-intervention |
| **Patty et al. (2018)**  **(The Netherlands)** | **Intervention:** Information and communication technology (ICT) training (including computer, iPhone, iPad and digital assistant devices training) in visually impaired adults.  **Comparator:** No control, comparisons with baseline measures. | **Setting:** Unclear. Patients undergoing rehabilitative eye care at two large eye care providers were enrolled between July 2014 and January 2015.  **Mode of delivery:** Not clear.  **Duration/intensity:** Training was tailored to each individual’s needs which meant training durations varied between participants  **Intervention details:**   - The ICT training included computer training (e.g. use of Word, the Internet and email) and training sessions on the use of iPhones, iPads and digital assistant devices. - At the rehabilitative eye care centres ICT training was a part of standard rehabilitative care - Data were collected pre training, post training and three months post training. |
| **Quialheiro et al. (2023)**  **(Portugal)** | **Intervention:** The OITO (Oficinas de Inclusão Tecnológica Online, “Workshops for Online Technological Inclusion”) project. A digital and health literacy digital inclusion project  **Comparator:** No control, comparisons with baseline measures. | **Setting:** In-person workshops, locations varied according region and workshop provider  **Mode of delivery:** 8 group workshop sessions delivered on 2 alternate days of the week lasting 1.5 hours per session  **Duration/intensity:** Not stated.  **Intervention details:**   - Sessions were divided into 45 minutes of digital activity, 10 minutes of physical activity, 30 minutes of digital activity, and 5 minutes of exchanging experiences in a “conversation circle - Participants used a mobile device, smartphone, or tablet, according of their choice. The workshops were led by an experienced trainer in teaching older adults how to use technology. One or two monitors further aided in assisting the OITO project participants - A brief physical activity moment consisted of standing exercises to stimulate circulation and balance training. - Data were collected pre- and post-intervention, and at 1-month follow-up. |
| **Seaton et al. (2023)**  **(Canada)** | **Intervention:** The Gluu Essentials digital skills training program**.** A digital skills training programme to support tablet use in older adults from rural areas.  **Comparator:** No control, comparisons with baseline measures. | **Setting:** Session were mainly provided from a distance (self-directed with telephone support) however some organisations delivered in-person support in small groups  **Mode of delivery:** 12 lessons delivered either in-person or remotely, e.g. via telephone.  **Duration/intensity:** No set duration, providers were free to deliver the programme how it worked best for participants and staff.  **Intervention details:**   - Twelve lessons were included in the Gluu Essentials training program, beginning with (1) the basic features of the tablet followed by (2) an introduction to the touch screen and gestures, (3) settings and tablet care, (4) the camera app, (5) email as well as Gmail or email app, (6) managing contacts, (7) find what is needed online, (8) how to download apps, (9) using the calendar app, (10) digital security basics, (11) Facebook basics, and (12) Zoom basics - The printed workbooks included a 96-page Gluu Essentials Student workbook tailored to each specific device - Sessions were delivered by volunteer coaches who were free to deliver the programme in the way that worked best for their learners - The Gluu Essentials training was provided free of charge; however, the devices were not provided by Gluu - Data were collected pre- and post-intervention. |
