# Supplementary material for: Effectiveness of Interventions for Addressing Digital Exclusion in Older Adults in the Social Care Domain: Rapid Review
Source: JMIR Aging. 2025 Dec 30;8:e70377. doi: 10.2196/70377 (PMC12826648; doi:10.2196/70377)
Supplement: Multimedia Appendix 4 [file aging_v8i1e70377_app4.docx]

| **Citation (Country)** | **Study Details** | **Participants & setting** | **Key findings** | **Observations/notes** |
| --- | --- | --- | --- | --- |
| Arthanat. (2021). [Promoting Information Communication Technology Adoption and Acceptance for Aging-in-Place: A Randomized Controlled Trial.](https://www.ncbi.nlm.nih.gov/pmc/articles/PMC7255945/pdf/nihms-1541973.pdf) *J Appl Gerontol.* 40(5): 471–480.  USA | **Study Design:**  Randomised controlled trial (RCT).  **Intervention:**  Home-based individualised inter-generational ICT training programme called the Individualised Community and Home-based Access to Technology Training (i-CHATT)  **Comparator:**  Control participants were older adults that did not receive any ICT training and participated in scheduled data collection under the premise of a longitudinal study on trends with ICT use.  **Study aim:**  To verify and report on the effect of a home-based individualised inter-generational ICT training program to facilitate ICT use and adoption, and self-reported independence among older adult trainees.  **Data collection method and dates:**  Data were collected using questionnaires which measured technology adoption, social participation, health and wellbeing. To measure changing attitudinal dispositions (acceptance) toward ICT, the Survey of Technology Use (SOTU) was used. To verify participants’ self-reported independence in activities relevant to ICT, the authors developed a 22- item questionnaire.  No dates were stated, however, data were collected in 6-month intervals at 6-, 12-, 18- and 24-months.  **Outcomes reported:**   - Technology adoption (56 activities for ICT use and 10 outcomes for frequency of use measured) - Self-reported independence (10 activities measured) - Technology acceptance (9 questions about attitudes) | **Sample size:**  97 participants (48 in intervention and 49 in the control group).  **Participants:**  Older adults (65 years and older) in demographic cohorts known to under utilise ICT, from small and rural towns.  **Setting:**  Delivered at participant’s homes. | **Primary Findings:**  **Technology adoption**  Both the training and control groups performed around 28 activities each month on average at the six-month follow-up almost the same as in baseline (27).  The training group maintained a more increasing trend during the remaining follow-up points ending at 36 activities compared to about 30 by the control group. The group X time interaction for the overall range of ICT use was found to be significant [F (4, 1) =2.5, *P*=.04, ηp2=.03]. Changes in the range of activities in specific categories were also analysed. No distinct trend or group X time interactions were evident for the range of five activities for family connections [F (4, 1)=.36, *P*=.8, ηp2=.004]. For the nine activities on social participation, the training group sustained an increasing linear trend higher than the control group, but the interaction was not statistically significant [F (4, 1)=1.75, *P*=.6, ηp2=.007]. Nevertheless, there were significant group X time interactions with a steadily increasing trend for participants in training group for 17 IADLs [F (4, 1)=3.4, *P*=.02, ηp2=.04], 11 health management activities [F (4, 1)=3.8, *P*=.006, ηp2=.04] and the 12 leisure activities [F (4, 1)=2.6, *P*=.04, ηp2=.03].  The training group performed 27 activities at a higher frequency trend in comparison to the control group across the five time points from baseline to two years, but the group X time interactions were not significant for all of them. Findings on the group X time interactions for the top ten activities that the training group performed at a higher frequency than the control group showed that seven out of the ten activities have significant interactions and the rest were approaching significance (p<0.1) the effects were small to medium. The control group performed better on frequency on six activities with none being significantly higher than the training group.  **Self-reported independence**  For 11 out of the 15 activities, the training group maintained higher trend in ratings over the control group following the ICT training. Significant group X time interactions were found in six of these activities: shopping [F (4, 1)=2.9, *P*=.02, ηp2=.036], engaging in volunteer activities [F (4, 1)=4.4, *P*=.002, ηp2=.05], finding new leisure activities [F (4, 1)=4.2, *P*=.002, ηp2=.05], maintaining connections with family [F(4,1)=2.8, *P*=.03,ηp2=.03], staying connected with your extended family [F(4,1)=2.7, *P*=.03, ηp2=.03] and initiating contacts and friendships [F (4, 1)=2.8, *P*=.02, ηp2=.03].  **Technology acceptance**  For the nine questions on predisposition to technology from the Survey of Technology Use (SOTU), the responses of the participants in the training group were noticeably positive compared to those in the control. Following the ICT training, older people in the training group (compared to those in the control) expressed that “technology experiences are satisfying” [F (4, 1)=3.5, *P*=.007, ηp2=.04], “technologies are encouraging” [F (4, 1)=2.4, *P*=.05, ηp2=.01], “I’m comfortable with technology [F (4, 1)=3.6, *P*=.009, ηp2=.04], and “I feel good around technology” [F(4,1)=2.3, *P*=.05, ηp2=.02]. |  |
| Castilla et al. (2018). [Teaching digital literacy skills to the elderly using a social network with linear navigation: A case study in a rural area](https://www.sciencedirect.com/science/article/abs/pii/S1071581918302672). *International Journal of Human-Computer Studies.* 118, 24-37.  Spain | **Study Design:**  Observational, uncontrolled study with repeated measures.  **Intervention:**  Eight standardised sessions focused on an online social network (Butler 2.0), delivered in person in groups of up to 6 people.  **Comparator:**  Baseline measures (pre-post).  **Study aim:**  To test a social network consisting of multiple applications with linear navigation as a digital literacy method for the elderly in rural areas.  **Data collection method and dates:**  Validated surveys, focus groups, dates not stated. The questionnaire on opinions about ICTs in general was designed ad hoc for this experiment, and consists of three items with a 5-point Likert scale. Modified questionnaires were used to measure usability and acceptability (the system usability scale).  **Outcomes reported:**   - Opinion about ICTs in general - Usability and acceptability items - Satisfaction with use - Recommendation of the system - Intended Use - Preference - Qualitative outcomes from focus groups | **Sample size:**  46 participants.  **Participants:**  60-76 years old from rural areas with heterogeneous previous experience with ICTs.  **Setting:**  Delivered at an Elderly Leisure Centre. | **Primary Findings:**  Regarding the differences between users with low and high ICT experience, there were differences in only one variable, perceived usefulness of Butler, t(44)=2.12, *P*=.040. After the first session using the system, users with less experience with ICTs rated a lower perceived usefulness than users with high experience. We then conducted repeated-measures ANOVAs in order to find out whether participants who were low and high on previous experience with ICTs behaved differently while using Butler. Again, the interaction effect was statistically significant only for perceived usefulness of the system F(1, 44)=4.277, *P*=.045. Users with low ICT experience began with a lower score on perceived usefulness than users with high ICT experience, and the use of the system resulted in increased perceived usefulness for low ICT experience users. After session 5, perceived usefulness was equal in both groups (A t-student test revealed no differences in perceived usefulness after session 5; t(44)=1.150, *P*=.257). Because no other differences were found due to previous experience with ICTs, the remaining analyses were performed with the entire sample. After the first session, all usability and acceptability items exceeded the midpoint of the scale (i.e., 2), placing Butler in the positive area of the scale on all variables, i.e. between "Neither agree nor disagree" and "Strongly agree". Regarding the way users felt when interacting with the system, we found that all the items also exceeded the midpoint of the scale, placing Butler in the positive area of the scale on all the variables In addition, after the first use of the system, 83% of the users said they would recommend Butler to other people of the same age, 2% said they would not, and 15% said they did not know if they would recommend it. No significant differences were found between the first and fifth sessions on perceived ease of use and usefulness and how the users felt while using the system. That is, users perceived the same ease and usefulness of the system, and they felt the same way as they did in the first lesson. The repeated use of the system significantly improved confidence, interest, and satisfaction with the system, and the sense of self-efficacy with regard to computer use. The effect size for these variables was small to medium. After five sessions of Butler use, the number of users who would recommend the system increased to almost the entire sample.  Regarding the users’ attitude toward new technologies in general, after the eight sessions of using the Butler 2.0 system, there was an improvement in all variables: Improvements in response to “How do you generally feel when using new technologies?” were not statistically significant (p>0.5). Answers to “To what extent do you feel capable of using new technologies?”, did improve significantly (*P*=.012), as were they to “To what extent are you interested in using new technologies?” (*P*=.003).  After completing the eight sessions of Butler use, 96% of the users expressed the intention to continue to use the system in the future, compared to 2% who expressed no intention to use it, and 2% who were unsure.  82.6% of the sample had received some kind of training in the use of ICTs. Given that previous experience was with hypertextual navigation systems, we asked about their preference regarding previous systems and Butler as a learning ICT method. 60.5% preferred the Butler method Perceived ease of use was one of the major reasons they preferred Butler as their ICT learning method. However, this variable was not important when choosing other ICT learning methods. The main reasons to prefer other systems were: “Other systems are more open” or “more realistic”. |  |
| Choi and Park (2022). [IT Humanities Education Program to Improve Digital Literacy of the Elderly](https://files.eric.ed.gov/fulltext/EJ1362299.pdf). *Journal of Curriculum and Teaching* 11,5, 138-145.  South Korea | **Study Design:**  Quasi experimental study  **Intervention:**  An educational programme which combines a decision tree with a game that allows the elderly to acquire IT knowledge. Consisted of 10 sessions in groups.  **Comparator:** General Internet and digital device use education (no detail about this intervention provided).  **Study aim:**  To test the effectiveness and evaluate satisfaction of an educational IT programme to improve digital literacy in the elderly (60+).  **Data collection method and dates:**  Satisfaction was assessed using a 15-question Likert survey, digital literacy was assessed using an edited version of the digital literacy competency self-diagnosis evaluation tool. Dates not stated.  **Outcomes reported:**   - Digital literacy, recognition & behaviour - Satisfaction with educational programme | **Sample size:**  42 participants (23 intervention 19 comparative group).  **Participants:**  Older adults (60 and older) who directly participated in the educational programme.  **Setting:**  Delivered at an education centre. | **Primary Findings:**  **Digital literacy, recognition and behaviour**  Digital literacy capabilities improved in the experimental (*P*<.05). The digital literacy capability was improved in the comparative group, but it was not statistically significant (*P*>.05).  The pre-average of the experimental group in recognition was significantly improved from 2.45(±0.55) to 3.02 (±0.64) (*P*=.012) after the intervention. The recognition area of the comparison group increased by 0.17 from a prior average of 2.34(±0.47) to 2.51(±0.35), but it was not statistically significant (*P*>.05).  In the behavioural area, the experimental group increased statistically significantly from a pre-average of 3.16(±0.66) to a post-average of 3.67(±0.59) (*P*=.001). The comparison group increased from a pre-average of 3.10(±0.81) to a post-average of 3.32(±0.34), but it was not significant (*P*>.05).  **Satisfaction**  Overall satisfaction was 4.13(±0.65). In detail, educational content was 4.22(±0.64), educational activity was 3.94(±0.76), and educational material was 4.05(±0.69). Male satisfaction (4.15±0.67) was higher than women's (3.98±0.77). In addition, the average satisfaction score of subjects aged 60 to 65 was 4.23(±0.59), and those aged 65 to 69 were lower at 3.58(±0.85). | *The educational program developed in this paper was delivered to the experimental group and the general Internet and digital device use education was conducted with the comparative group.* |
| Czaja et al. (2018). [Improving Social Support for Older Adults Through Technology: Findings From the PRISM Randomized Controlled Trial](https://watermark.silverchair.com/gnw249.pdf?token=AQECAHi208BE49Ooan9kkhW_Ercy7Dm3ZL_9Cf3qfKAc485ysgAAA2IwggNeBgkqhkiG9w0BBwagggNPMIIDSwIBADCCA0QGCSqGSIb3DQEHATAeBglghkgBZQMEAS4wEQQMnWMqZlJNNgoGvwAgAgEQgIIDFUpj7e9f3kstQo_eDnhUcRW1hTVPs_AWavln0kDXALmbslOA1e4M6iGa-tc5_7wteolyXtaqnnUMbbJ1oeoJC7sDkiLsPAwo3T3cY-Z0x0r0AY44kH591OmRiYP9e0nLhyP_uUcZnsbNeLCIyk43RjWxiaAwUmKjLLccSjOsoqw18FngFz5Y4sahA7NxLGFiXsWUrqzmf25Gl8YLamrl1ib5ryBZJYeRDUdtoD0zf_-Dq54NXkG25g-g27Sv2jUzR2umO9f5EeX5B6VKKFwhmKq_iiazL4qe6MWtwNMEh_aRSVX8Jii38HK0KFI6wTjFtW-sgMSU0wG75FnEzqFftLkF7HoRZcayjjBxKOUR2uwT-L8FUv2Pa-3o6KPob_HQsp966UrpwFpZbYJDfyx6-q5V8UvsC7EHEu6BgQCW3IIuw3XxTevKzATbtp1KyUmJMyClirfdaHjOEUpHd8t-FU4pVpL5gFU-kXxnJNSufD7upvUu8td1pqD2n1tdKJVAnx7-XQacacIAiY2seiC14iUDGm4-lkjHxDu0X8TuCdwQVwpltu6BU7meQvSEmZuENl5H4laktjG-HuSghL_1PXFNfo3vbZ5LI8yUv2sn2A2sRp0_S4wFM6M2VVqA-fPiQobzYQzZb5YJWiTucP8rzzh88x6HgSfPyHPd_HQGzH_gJQQmumjbCopZcbknobkjg5okXVLaHvzceaglwLVmQQVQoxvSiZstabNvUEPD4zE5XFC6ZQAXdmNtPvCxtNzR6p0M6_cUFnjPI3tIhwU26eQYEYw4Tew7AMl_7udDwFbtmgvYiZAIocJ0RN4DJGXmEJd3_F5T8XprH-Kx8uQVNnvCIk7QcxfOentDKg3lQZSYWH44k0ATVoKcu2FxhrcpiOsdyPiPnevYut4i-njFq53LH4T8AuPDjuQe11srOVxt6Qdj2W651us1WPq03ffPA_5MJNUIoQ64ShkyUDi3RhbNuietnOMvnALQiL5Tb-1hp9vLYioa0qJ-bw7T2OH8kkFcv65KpvSdQ7luWsAkyhlb9PmxjQ). *Gerontologist* 58,3, 467–477.  USA | **Study Design:**  RCT  **Intervention:**  The Personal Reminder Information and Social Management (PRISM) system is a specially designed computer system for older adults, which includes a software application and a robust support system with training and instructional support. PRISM participants received a Lenovo “Mini Desktop” PC with a keyboard, mouse (or trackball for those who were unable to control a mouse), a 19″ LCD monitor, the PRISM software application, and a printer. They were provided with access to internet. It also included an annotated resource guide, a dynamic classroom feature, a calendar, a photo feature, E-mail, games, and online help. Resources included information on local and national organisations, services such as transport and meals, and educational offerings. Participants also received home visits, check-in calls, a help card and access to a technical help line.  **Comparator:**  Participants in the comparator (Binder) group received a notebook that contained paper content similar to that contained in the intervention.  **Study aim:**  To evaluate the impact of a specially designed computer system for older adults.  **Data collection method and dates:**  Data were collected using the Life Space Questionnaire, the Test of Functional Health Literacy in Adults, Wide Range Achievement Test (WRAT), and measures of cognitive abilities such as working memory and processing speed, System Evaluation questionnaire. Data were collected at baseline, 6- and 12-months, dates not stated.  **Outcomes reported:**  Primary outcomes included:   - Social isolation - Loneliness - Perceived social support - Social Network Size - Perceptions of quality of life - Perceived vulnerability - Changes in health-related quality of life and wellbeing   Secondary outcome measures include:   - Computer proficiency - Attitudes toward technology - Perceptions of the usefulness and usability of PRISM (the intervention) | **Sample size:**  300 participants (150 in intervention, 150 in the Binder group)  **Participants:**  English speaking older adults (65 and older) who had at least 20/60 vision with or without correction, and could read at the 6th grade level, at risk for social isolation who lived independently in the community; minimal computer/internet use, not employed or volunteering more than 5 hr/week, or spending more than 10 hr/week at a senior  centre or formal organisation.  **Setting:**  Delivered at participant’s homes. | **Primary Findings:** Relative to those in the Binder condition, PRISM participants reported greater increases in computer comfort at 6 months (b=−1.68; *P*<.001; effect size = .39; 95% CI: −2.57 to −.78) and 12 months (b=−2.32; *P*<.001; effect size = 0.53; 95% CI: −3.22 to −1.41); greater increases in computer interest at 6 months (b=−1.52; *P*<.001; effect size = .46; 95% CI: −2.26 to −.79) and 12 months (b=−.99, *P*<.01, effect size = .30; 95% CI: −1.74 to −.25); and had greater increases in computer efficacy at 6 months (b=−1.29; *P*<.001; effect size = .41; 95% CI: −2.01 to −.57) and 12 months (b=−.94; *P*<.02; effect size = .30; 95% CI: −1.67 to −.22). Those assigned to PRISM also demonstrated a greater increase in computer proficiency at 6 months (b=−6.37, *P*<.001; effect size = 1.11; 95% CI: −7.39 to −5.35) and 12 months (b=−7.06, *P*<.001; effect size = 1.23; 95% CI: −8.08 to −6.03).  **Technology Acceptance Questionnaire and System Evaluation**  Most PRISM participants found PRISM useful in their daily life (82%), indicated that PRISM made their life easier (80%), improved their daily life (84%), and enabled them to accomplish tasks more quickly (73%). They also found PRISM easy to use (88%) and easy to become skilled at using PRISM (80%). In fact, 60% indicated that they felt comfortable using PRISM within 1 week. Most found the E-mail feature valuable (85%), using the Internet valuable (82%), the Classroom feature valuable (80%), and the Games as valuable (77%). Only 57% found the Photo feature valuable and 51% found the Calendar valuable. Of those who received the Binder, 89% found the information and tips valuable and 87% found the community resource information valuable and about half (58%) found the Calendar valuable. In contrast to those who received PRISM, only 61% thought the games were valuable. PRISM participants found it easier to communicate with family and friends relative to those who received the Binder (82% vs. 47%) and engage in hobbies and play games (82% vs. 52%). Participants in both conditions reported that it was easier to look up community information (78% vs. 73%) and health information (82% vs. 80%). Almost all of the PRISM participants (93%) found PRISM enjoyable to use and 88% of those receiving the Binder found it enjoyable to use. However, those in the Binder condition were more satisfied with the in-home training they received (90% vs. 82%). | *The secondary outcomes reported appear to address the research question* |
| Elbaz et al. (2023). [Evaluation of a virtual 4-week digital literacy program for older adults during COVID-19: a pilot study](https://www.tandfonline.com/doi/full/10.1080/03601277.2023.2268499). Educational Gerontology,  Canada | **Study Design:**  Quasi-experimental (Pilot study).  **Intervention:**  Four-weekly digital literacy intervention programme offered through the Zoom conferencing platform. Sessions were delivered by a trainer and two co-facilitators and covered a range of skill-based topics, including digital safety and security. On a handful of occasions (3–4 times), the trainer met with the participants in one-on-one meetings to go over concepts on a request basis.  **Comparator:**  Baseline (pre-post)  **Study aim:**  To evaluate the effectiveness of a 4-week digital literacy programme to train older adults on key skills related to navigating their computer (e.g. sending e-mails or traversing the web).  **Data collection method and dates:**  Data were collected using the Computer Proficiency Questionnaire (CPQ). Dates of data collection not stated.  **Outcomes reported:**   - Average computer proficiency score - Participant perceptions | **Sample size:**  5 participants  **Participants:**  A convenience sample of elderly adults (65 years and older, 1 male and 4 females) from a large-scale telehealth program. All older adults were required to have an Internet connection and a digital device that could connect to the Internet (e.g. a computer, mobile phone or tablet with an Android or iOS operating system).  **Setting:**  Delivered remotely online via Zoom. | **Primary Findings:**  **Computer proficiency score**  The scores of participants mean CPQ scores were higher after the 4-week intervention, compared to pre-intervention scores [17.72 (±1.94) vs. 13.24 (±2.40), t(4)=−8.910, *P*<.001)], suggesting that overall digital literacy seemed to improve over the intervention period. Mean CPQ scores post intervention were also significantly higher compared to pre-intervention for the computer basics [3.97 (±0.45) vs. 3.23 (±0.60), t(4)=−5.880, *P*=.004)], communication [3.36 (±0.38) vs. 2.40 (±0.48), t(4)=−8.353, *P*=.001)], and Internet subscales [3.63 (±0.16) vs. 2.71 (±0.48)], t(4)=−4.257, *P*=.013)], suggesting that participants may have gained added knowledge and confidence regarding these aspects after the intervention. No significant differences were found between pre and post intervention scores in printing, scheduling, and multimedia subscales (*P*>.05), indicating that the intervention had no effect on these topics. Overall, the findings indicate that participants perceived digital literacy seemed to improve through the 4-week intervention.  **Participant perceptions**  General feedback from the participants were initially related to the speed in which the trainer discussed the concepts. Additionally, at times some participants mentioned being confused as to why certain applications appeared different (i.e., had a different name than what the trainer was showing, different icons, or the user interface was not the same). Overall, participants all mentioned appreciating the digital literacy training as well as the accessibility, and patience of the trainer and facilitators. |  |
| Fields et al. (2020). [In-Home Technology Training Among Socially Isolated Older Adults: Findings From the Tech Allies Program](https://escholarship.org/uc/item/0qm1k9gh). *Journal of Applied Gerontology.* 1-11.  USA | **Study Design:**  RCT  **Intervention:**  Tech Allies Eight-weekly one-to-one digital training sessions incorporated into the Little Brothers - Friends  of the Elderly (LBFE) volunteer-based friendly visitor programme. Participants each received a tablet, broadband access and a certificate of completion at the end of the programme. Volunteers provided eight weekly, in-home iPad lessons. Participants each received a learner booklet, outlining curriculum topics by week, including step-by-step visual guides and practice exercises.  **Comparator:**  A 2-month waitlist group served as the control group  **Study aim:**  To evaluate the effect of providing digital devices (tablets), internet access (through a discounted senior broadband program), and in-home 1:1 technology training to isolated older adults, on participants’ loneliness, perceived social support, and technology use.  **Data collection method and dates:**  Data were collected using questionnaires and interviews. To measure technology use, participants were asked whether or not they used the internet at least occasionally and participants self-reported confidence. Data collection dates not stated but sample recruited between July 2017 and December 2018.  **Outcomes reported:**   - Self-reported loneliness - Social support - Technology use - Confidence at baseline and follow-up | **Sample size:**  83 participants, 44 in the intervention arm (27 with complete post-training survey data) and 39 in the waitlist arm (30 with complete 2-month waitlist survey data).  **Participants:**  Older adults (65 and older or 60 and older with a disability), who received fewer than two social visits each month, (socially isolated) were English-speaking, and expressed a need for in-depth technology training.  **Setting:**  Delivered at participant’s homes. | **Primary Findings:**  **Technology use**  There were significant improvements in technology use from baseline to 2 months within the intervention arm (baseline = 33% no internet or email use, 2-month = 0% no internet or email use, *P*=.004). There was no change over time within the waitlist arm (baseline = 53% no internet or email use, 2-month = 60% no internet or email use, *P*=.63).  **Confidence**  Within the intervention arm, there was improvement in confidence in digital skills (baseline = 52% little to no confidence searching for information online and using email, 2-month = 35% little to no confidence, *P*=.13) and no change in the waitlist arm (baseline = 76% little to no confidence, 2-month = 77% little to no confidence, *P*=1.0).  **Results from logistic regression:**  Participants had greater odds of reporting technology use, OR 91.20 the (95% CI: 11.02 to +Infinity) and odds of reporting confidence in their technology skills OR 8.99 (95% CI: 1.55 to 96.57) compared with those in the waitlist group. Results were adjusted for baseline technology use and e technology confidence in searching for information and email use.  **Participant perceptions:**  Many participants expressed feeling more confident navigating technology and less scared of digital devices.  Participants also provided feedback on the program. This included a desire for more total sessions and more frequent sessions with their volunteer instructors to provide additional opportunities for repetition and practice as well as more time to progress to advanced topics. While some participants did not regularly use the written materials provided as part of the program, others felt that more detailed visual and written instructions would be helpful. Participants also highly valued having someone to guide them and answer their questions about the tablet. Some expressed a desire for more structure from their volunteer instructor, while others appreciated the highly customised, participant-driven approach. For many participants, their personality match with their instructor was central to their program experience. |  |
| Gadbois et al. (2022). [Findings From Talking Tech: A Technology Training Pilot Intervention to Reduce Loneliness and Social Isolation Among Homebound Older Adults](https://academic.oup.com/innovateage/article/6/5/igac040/6598241). *Innovation in Aging.* 6;5,  USA | **Study Design:**  Quasi-experimental (Pilot study).  **Intervention:**  The Talking Tech intervention was a 14-week, volunteer-delivered programme whereby participants were provided training and assistance by “TechMates”, delivered one-to-one and embedded within and delivered by a home-delivered meals programme.  **Comparator:**  Baseline (pre-post).  **Study aim:**  To report findings from a pilot intervention designed to reduce loneliness and social isolation among homebound older adults by providing one-on-one, in-home technology training to promote digital literacy and participation in a virtual senior centre.  **Data collection method and dates:**  Data were collected using questionnaires and interviews.  Questions modelled after the Technological Environment section from Round 7 of the National Health and Aging Trends  Study were used to assess technology use No data collection dates stated, however, the Talking Tech programme ran between October 2019 and May 2020.  **Outcomes reported:**   - Loneliness - Social isolation - Technology use (internet use, email and text messaging use, smartphone, tablet, and computer ownership, and well as online information-seeking behaviour) - Health satisfaction - Quality of life - Self-rated health - Activities of daily living - Independent activities of daily living needs - Depression | **Sample size:**  21 participants  **Participants:**  Older adults (60 and older) who were homebound or had difficulty leaving their homes independently.  **Setting:**  Delivered at participant’s homes. | **Primary Findings:**  **Technology use**  There was a trend towards some increased use of technology, but none of the results were statistically significant. Sent email or text messages most days in past month: At baseline, 14 (78%), post intervention, 13 (72%) *P*=1.0. Used internet in past month: At baseline, 11 (61%), post intervention 13 (72%) *P*=.27.  Online activity engagement score:  At baseline, 1.44 (1.69), post intervention 1.89 (1.84) *P*=.44.  Shopping in past month, at baseline 4 (22%), post-intervention 4 (22%). Order or refill prescriptions in past month, at baseline 3 (17%), post-intervention 5 (28%).  Pay bills or banking in past month, at baseline 6 (33%), post-intervention 2 (11%). Social networking sites in past month, at baseline 7 (39%), post-intervention 9 (50%)  Contact medical provider in past month, at baseline 2 (11%), post-intervention 6 (33%). Health insurance matters in past month, at baseline 2 (11%), post-intervention 3 (17%). Sought health information in past month, at baseline 9 (50%), post-intervention 10 (56%).  **Participant perceptions:**  Nearly all participants reported having a positive experience with Talking Tech. TechMate reports indicated that 15 of the 16 participants for whom reports were received were “very interested” in the program and “excited” to learn how to use the tablet. A few participants and TechMates suggested including more training sessions to accommodate participants with a slower learning pace or for those starting with limited technology knowledge. Alternatively, others with more computer experience described that aspects of the module content were too basic. |  |
| Garcia et al. (2022).  [Impact of an educational program to improve older adults’ digital skills.](https://www.researchgate.net/publication/370737430_IMPACT_OF_AN_EDUCATIONAL_PROGRAM_TO_IMPROVE_OLDER_ADULTS'_DIGITAL_SKILLS) *Egitania Sciencia*, 9-31  Multiple countries including Latvia, Poland, Portugal and the United Kingdom. | **Study Design:**  Quasi-experimental study.  **Intervention:** The Erasmus+ project ICTskills4All examined  three different educational approaches (intergenerational, peer-to-peer and online) for improving digital skills in older adults were compared.  **Comparator:**  Three different intervention delivery methods were compared (see above)  **Study aim:**  To analyse the impact of three different educational approaches (intergenerational, peer-to-peer and online) on older adults’ digital skills in different European countries (Latvia, Poland, Portugal and the United Kingdom) participating in the Erasmus+ project ICTskills4All.  **Data collection method and dates:**  Data were collected using the Digital Skills Self-Assessment Questionnaire and interviews.  **Outcomes reported:**  Digital literacy:   - Information and data literacy (I&DL) - Communication and collaboration (C&C) - Digital content creation (DCC) - Safety (S) | **Sample size:**  39 participants (30 for the two in-person delivery formats, 9 in the online group)  **Participants:**  Older adults aged 55 years and above from Latvia, Poland, Portugal and the United Kingdom, 66.7% of participants across the countries were aged 65-74. Participants had their own computers.  **Setting:**  Delivered in-person and online. | **Primary Findings:** There was no significant difference comparing pre and post scores in the ND (motivation) domain in any of the approaches, which showed that none of the formats was effective in improving participants’ motivation.  The online course was more effective in improving digital content creation skills, being the only one that showed a significant difference in this domain.  The intergenerational and peer-to-peer groups did not show significant differences between them. Both groups, however, showed a difference when compared to the online approach in the information and digital literacy, communication and collaboration, and safety domains.  Regarding the questions of the four identified domains, the peer-to-peer and intergenerational programs were effective in improving the skills evaluated in 45 questions: all 23 competences in I&DL (100%); all 9 competencies in C&C (100%); and 13 of the 14 competences (93%) in S. In the online format, the course proved to be effective in improving the skills evaluated in 35 (71.4%) questions: 14 out of 23 (61%) in I&DL; 5 out of 9 (56%) in C&C; all 14 skills (100%) in S; and all 2 skills in DCC (100%).  Peer-to-peer and intergenerational courses were more effective in improving I&DL and C&C skills (100% effective in both domains, compared to 61% and 56% effective, respectively, in online courses).  **Peer-to-peer**  **Information & data literacy**: Significant improvements from baseline (2.09 to 3.72, *P*=.0001) **Communication and collaboration:** Significant improvements from baseline (1.78 to 3.22, *P*=.0001) **Safety:** Significant improvements from baseline (1.93 to 3.29, *P*=.0001) **Motivation:** No significant change from baseline (4.0 to 4.5, *P*=.606) **Digital content creation:** No significant change from baseline (3.25 to 4.0, *P*=.501)  **Intergenerational**  **Information & data literacy**: Significant improvements from baseline (2.41 to 3.91, *P*=.001) **Communication and collaboration:** Significant improvements from baseline (2.06 to 3.44, *P*=.01) **Safety:** Significant improvements from baseline (2.0 to 3.43, *P*=.012) **Motivation:** No significant change from baseline (4.0 to 4.0, *P*=.755) **Digital content creation:** No significant change from baseline (4.0 to 3.75, *P*=.977)  **Online**  **Information & data literacy**: Significant improvements from baseline (4.13 to 4.83, *P*=.019) **Communication and collaboration:** Significant improvements from baseline (3.67 to 4.78, *P*=.05) **Safety:** Significant improvements from baseline (3.50 to 4.57, *P*=.0001) **Motivation:** No significant change from baseline (4.0 to 5.0, *P*=.387) **Digital content creation:** Significant improvement from baseline (3.0 to 4.0, *P*=.004)  **P1. Comparison between peer-to-peer and intergenerational**  No significant differences in outcomes for any domain.  **P2. Comparison between peer-to-peer and online**  Significant differences in information & digital literacy, communication and collaboration, and safety (appear in favour of peer-to-peer but not very clearly reported). No significant differences in ND (motivation) or digital content creation.  **P3. Comparison between intergenerational and online**  Significant differences in information & digital literacy, communication and collaboration, and safety (appear in favour of intergenerational but not very clearly reported). No significant differences in ND (motivation) or digital content creation. | At baseline, each group were comparable for motivation (ND), and digital content creation. However, there were baseline differences in I&DL, C&C and S domains, online participants had more skills at the beginning of the course than the peer-to-peer and intergenerational participants. |
| Holguin-Alvarez et al. (2020). [Digital competences in the social media program for older adults in vulnerable contexts.](https://repositorio.ucv.edu.pe/bitstream/handle/20.500.12692/70211/AC_Holguin_AJA-Manrique_AG-Apaza_QJ-Romero_HR.pdf?sequence=1) *International Journal of Scientific and Technology Research,* 9 (5), 228-232.  Peru | **Study Design:** Quasi-experimental study  **Intervention:** Social media programme designed to increase digital skills in communities at risk of social exclusion. The experimental pedagogical program was developed during 50 learning activities, based on the use of Facebook, WhatsApp, Twitter and Gmail were applied as essential elements of virtual communication. Session lasted 30 minutes. The experimentation was manipulated in four stages for each activity: 1) Dynamic familiarisation; 2) Thematic introduction; 3) Network Registries; and 4) Digital interactivity.  **Comparator:** No intervention  **Study aim:** To verify the effects of imparting activities of a program based on Social Networks in the digital competences of the elderly.  **Data collection methods and dates:** The instrument used to gather information on digital competences was the Questionnaire on Digital Competencies of University Students and for Non Students (adapted version); which consisted of 31 items, with four scales dedicated to the evaluation of the effectiveness in digital competence. This questionnaire was administered before and after the intervention. Also, an open response form was used to record the opinions of some participants regarding the usefulness they felt in their digital competences after participating in the experimental program. Dates of data collection not stated.  **Outcomes reported:**   - Digital competencies | **Sample size:** 40 participants (20 in intervention and 20 in the control group)  **Participants:** Older adults in vulnerable contexts (Low SES) (81 -92 years) who regularly attended The House of the Elderly, located in a central district of the city of Lima, in Peru.  **Setting:** Delivered in the community. | **Primary Findings:**  The results regarding the digital competences variable before the application of the program (pre-test measurement), reported equitable measures between the control group (M(y)=31,5; SD. (y)=0.42) and experimental group (M(x)=46,3; SD. (x)=1.03). In this analysis, the comparison of group averages did not report statistical significance (t(34)=-1,264; sig.=2,56; *P*>.001). In relation to the digital competencies in post-test measurement (after the application of the program), average scores were obtained that differed from each other. The measures of the experimental group (M(x)=119,1; SD. (x)=.24) and the control group (M(y)=45,1; SD. (y)=1.06). Regarding the statistical analysis, the differences were corroborated with a degree of success of 99% (t(39)=-2,146; sig.=.000; *P*<.001). | This paper contains limited methodological information. Also, the study has been poorly translated in areas, these factors make it difficult to definitively extract all key details of the study. |
| Lee and Kim (2018). [Bridging the digital divide for older adults via intergenerational mentor-up.](https://journals.sagepub.com/doi/epub/10.1177/1049731518810798)*Research on Social Work Practice*, 29(7, pp.786-795.  USA | **Study Design:** Quasi-experimental study  **Type of intervention:** The Intergenerational Mentor-Up (IMU) programme contained guided learning opportunities for undergraduate students to teach older adults IT skills and reduce their social isolation. Six group IMU sessions were held where mentees were paired with student mentors who provided one-to-one support based on the specific needs of mentee. At the end of each session, everyone came together, so that the seniors could share their accomplishments with the entire group.  **Comparator:** Pre-post intervention comparisons  **Study aim:** To examine the effectiveness of Intergenerational Mentor-Up (IMU), an innovative intervention that engages college students in tutoring older adults, with regard to E-Health literacy and social isolation.  **Data collection methods and dates:** Data were collected using semi-structured face-to-face interviews administered by five research assistants prior to and after participating in the IMU. The interview used established questionnaires to determine eHealth literacy (eHEALS scale), attitudes towards computers/internet (Computer Efficacy subscale), technophobia (computer anxiety scale) and social isolation. The results of each scale were compared to pre-test measures. Dates of data collection not stated.  **Outcomes reported:**   - eHealth literacy - Technophobia - Self-efficacy - Social isolation - Interest in technology | **Sample size:** 55 participants  **Participants:** Older adults (65 and older), 63.6% were female and 36.4% were male. In addition, 56.4% were non-Hispanic Black and 38.2% were non-Hispanic White. The majority lived alone for a mean of 11.39 years. They had a mean of 2.65 chronic illnesses (SD=1.65). Judging from the number of life stressors, the IMU participants reported low level of stress.  **Setting:** Half of the sessions were offered at senior centres, and the half in housing facilities. The study took place in a South-eastern city that was ranked lowest for social mobility (low income areas). | **Primary Findings:**  *eHealth literacy*: at pre-test, eHEALS scores suggest that self-rated eHealth literacy, on average, was at a neutral (i.e., “undecided”) level, and the IMU senior mentees, on average, showed significant improvement at post-test (t=−5.89, *P*<.001, d=−.79). Senior mentees’ views about the Internet’s usefulness in helping them to make decisions about their health were significantly changed from pre- to post-test (t=−4.6, *P*<.001, d=−.62), as was their opinion about the importance of being able to access health resources on the Internet (t=−4.35, *P*<.001, d=−.59). At pre-test, IMU senior mentees expressed a lower level of willingness to use online health information (if someone taught them how to use the computer/Internet). By post-test, their levels of willingness (t=−7.99, *P*<.001, d=−1.08) significantly increased.  *Attitudes*: attitudes toward computers/Internet were measured with the efficacy and interest subscales. IMU senior mentees showed significant improvement in their self-efficacy (t=−8.36, *P*<.001, d=−1.13) and interest (t=−9.24, *P*<.001, d=−.25) in using computers/Internet at post-test.  *Technophobia:* Following the IMU training, senior mentees also felt confident about their skills in utilising computers/Internet (t=−3.69, *P*<.001, d=−.50). As a consequence, their anxiety toward technology decreased (t=2.65, *P*<.01, d=.36).  **Participant perceptions:**  Qualitative data revealed that individualised training, modifications, adaptations, and intergenerational interactions can decrease older adults’ anxiety and boost their confidence. Benefits perceived by IMU senior mentees were emerged into four major themes: communication tools, independent living, leisure activities, and intergenerational learning.    Communication tools  Following their IMU lessons, many of the older adults reported accomplishments, including sending a “first” text to a daughter and sharing photos with a grandchild via Facebook.    Independent living  Although the IMU senior mentees were not afraid of or unwilling to use technology and were able to acquire the necessary skills, many reported usability problems (e.g. difficulty with reading small fonts, difficulty of navigation) and associated frustration with the computer system due, in part, to the cognitive, perceptual, and motor skill demands that they required. Youth mentors were helpful in pointing out options for assistive technology, such as magnifiers, flashlight, and speech-to-text software as well as enlarging fonts.    Leisure activities  Youth mentors also helped a few of the older adults to explore online dating sites. Other popular activities were to search recipes and to get directions, the weather, and travel information.    Intergenerational learning  Both the senior mentees and students expressed that mutual learning was occurring in terms of technology skills, life experiences, and shared interests. The words used to describe this intergenerational experience included “awesome,” “happy,” “inspiring,” and “a real joy.” Almost all IMU senior mentees felt that working with their youth mentors made them “feel young.”. As the IMU program was winding down, several senior mentees asked whether they could have more technology classes. Almost all senior mentees asked for “more classes like this.” | Most participants (n=40) were internet and daily technology (n=30) users before the program commenced.  The programme was part of a university project aimed to improve undergraduates ability to mentor and communicate with older adults. |
| Lee et al. (2022a). [Effect of a digital literacy program on older adults’ digital social behavior: A quasi-experimental study.](https://www.mdpi.com/1660-4601/19/19/12404)*International Journal of Environmental Research and Public Health*, 19(19), p.12404.  South Korea | **Study Design:** Quasi-experimental study  **Type of intervention:** A digital literacy education programme to improve smartphone usage competency. Sessions ran once a week for six weeks and included topics such as, basic smartphone operation, sending text messages, taking and sharing photos, social app, search engine applications, and internet banking.  **Comparator:** A no intervention control was used. Statistical comparisons were made pre- vs post-intervention  **Study aim:** To assess the effects of digital literacy education on digital device usage and evaluate the positive effects of digital literacy education  **Data collection methods and dates:** Data were collected using questionnaires to assess participant characteristics, happiness, subjective health, depression, quality of life, self-efficacy and a cognitive function test. Self-efficacy was assessed using the 10-item General Self-Efficacy Scale (GSE) Dates of data collection not stated, however, the programme ran from 25 October 2021 to 3 December 2021.  **Outcomes reported:**   - Digital device usage - Depression - Happiness - Quality of life - Self-efficacy - Cognitive function | **Sample size:** 144 participants (62 in intervention and 62 in the control group). Post-intervention data was only record from 45 people in the experimental group and 36 people in the control group responded to the survey  **Participants:** Older adults (65 years and older) living in rural areas  **Setting:** Delivered across a total of five locations including Y University Healthy City Research Center, the Wonju Senior Center and small libraries. | **Primary Findings:**  The frequency of phone calls made using smartphones by the older adults in the experimental group increased significantly by 8.5% after education (t=1.934, *P*=.026 ). In contrast, no changes were observed in the control group.  No significant increase in taking photos was observed in either group after the intervention period (experimental group: *P*=.087; control group: *P*=.176)  A significant increase in ability to video record using a smartphone was observed in the experimental group (t/x^2^=4.493, *P*=.049, no improvement was shown for the control group (t/x^2^=.522, *P*=.5277)  Self-efficacy  Experimental group improved after the intervention but not significantly (57.1-58.1 *P*=.530), the control group increased significantly (55.6-60.1 *P*=.025). However when compared no significant difference was reported between groups ((t)-1.382 95%CI [−8.499, 1.494]; *P*=.169). | This intervention also evaluates the effects of digital literacy education on depression, happiness, quality of life, self-efficacy and cognitive function.  In relation to the discrepancy between the number of participants undergoing the program and the number of participants completing the final survey: the remaining participants were unable to participate on the last day due to COVID-19, and hence, the survey was not conducted. |
| Lee et al. (2022b). [Information communication technology use to improve eHealth literacy, technophobia, and social connection among community dwelling older adults.](https://www.researchgate.net/profile/Othelia-Lee/publication/359085432_Information_Communication_Technology_Use_to_Improve_eHealth_Literacy_Technophobia_and_Social_Connection_among_Community_Dwelling_Older_Adults/links/63640c9d2f4bca7fd02b3b17/Information-Communication-Technology-Use-to-Improve-eHealth-Literacy-Technophobia-and-Social-Connection-among-Community-Dwelling-Older-Adults.pdf) *Educational Gerontology,* 48(10), pp.445-457.  South Korea | **Study Design:** Pre-test-post-test non-equivalent control group design  **Type of intervention:** The Intergenerational Forum (IF). A 12-week, class educational programme providing guided instruction and intergenerational  exchange between youths serving as mentors and older adults serving as mentees. Classes consisted of 8–10 mentees. Mentees shared learning goals and IT issues, and were given group tutorials and individualised support.  **Comparator:** No intervention control group. Within-group pre-test and post-test comparisons were made  **Study aim:** To examine the outcome of a 12-week class focused on encouraging intergenerational exchange and mutual aid between college students and older adults.  **Data collection methods and dates:** For each participant, a pre-test was conducted by the research team a week prior to the IF classes and a post-test was done a week following the IF classes. Surveys were used to examine the participants’ e-Health literacy The e-Health Literacy Scale (e-HEALS), technophobia, adapted from the Computer Anxiety Scale (CAS) social isolation, and social capital in relation to older mentees’ processes of learning and mastering the ICT skills via IF classes. Study participants’ prior experiences of using the Internet were assessed, including frequency of Internet use, challenges faced, issues of accessibility, types of devices, and individual use patterns. Data collected between 2017 and 2019.  **Outcomes reported:**   - eHealth literacy - Technophobia - Usefulness of the internet - Importance of internet - Anxiety | **Sample size:** 104 participants (50 in intervention group and 54 in the control group).  **Participants:** Older adults (65 years and older) attending one of two senior centres, interested in learning about computers and the internet.  **Setting:** Two large senior centres in metropolitan Seoul, Korea | **Primary Findings:** The results did not show  a significant difference in the mean scores for the intervention and comparison groups in all study outcome variables (e-Health literacy, technophobia, social isolation, etc.).  *E-Health literacy*: Following the 12-week IF classes, older mentees improved in their eHealth literacy (t(49)=−4.23, *P*<.001, d=−.60). The IF participants also reported perceived greater usefulness of Internet during this period (t(49)=−3.50, *P*<.001, d=−.49).  *Technophobia:* Considering two dimensions of technophobia, anxiety toward technology was significantly reduced in IF group participants throughout the study (t(49)=−2.77, *P*<.01, d=−.39). Consequently, IF group participants reported an overall increase in the level of confidence in using technology (t(49)=−5.05, *P*<.001, d=−.71). Within the comparison group, significant changes were observed during the study period in the two outcomes of technophobia. Comparison group participants’ anxiety toward technology was reduced (t(53)=−2.04, *P*<.05, d=.28).  **Additional Findings:** Results of ANCOVA indicated that there was no statistically significant difference between the grouping conditions on the adjusted post-test means at the *P*<.05 level for all variables, except for confidence. The effect size partial eta squared measures for the variables with non-significant differences were .04 or below, which are considered small. The adjusted mean confidence post-test score for the intervention group (M=2.87, SD=.47, M_adj_=2.84) was significantly different from the comparison group (M=2.58, SD=.56, M_adj_ j=2.61) (F(1,101)=9.99, *P*=.002, η2p =.09), and the effect size partial eta squared of .09 (90% CI: .02 to .19]) indicated a small effect. | *Baseline differences in internet use was apparent between groups* (χ2(2)=27.34, *P*<.001). While all IF participants identified as current users of Internet, only slightly over half of the comparison group identified as current users (57.4%).  More males were represented in the IF group, whereas more females were represented in the comparison group (χ2 (1)=10.9, *P*<.001). |
| Ma et al. (2020). [Bridging the digital divide for older adults via observational training: Effects of model identity from a generational perspective.](https://www.mdpi.com/2071-1050/12/11/4555) *Sustainability*, 12(11), p.4555.  China | **Study Design:** Quasi-experimental study  **Type of intervention:** A video tutorial-based intervention. Participants were divided into three intervention groups that differed according to the model in the videos (a child model, young adult model, or older adult model). The groups received the exact same content, albeit delivered by a different model, which aimed to familiarise them with digital technology and allow them to perform essential tasks.  **Comparator:** Between groups assessment to examine the effect of different observational models and pre-post intervention comparisons  **Study aim:** To investigate the effectiveness of observational training through behaviour modelling in enhancing technology acceptance in older adults.  **Data collection methods and dates:**  Data were collected using a pre and post-intervention survey, and a technical quiz and post-training questionnaire.  Data collection dates not stated.  **Outcomes reported:**   - Self-efficacy - Outcome expectation - social connectedness | **Sample size:** 59 participants (21 in the child model group, 19 in the young adult model group, and 19 in the older adult model)  **Participants:** Community-dwelling Chinese older adults (60 and older) with little or no experience of using a tablet  **Setting:** The Salvation Army Chuk Yuen  Centre for Senior Citizens, in Hong Kong | **Primary Findings:**  *Pre vs post findings:*  For meta-cognitive outcomes (i.e., self-efficacy, outcome expectation, and social connectedness), when considering the whole sample, all scores significantly increased after the training.  Comparing the scores of the three meta cognitive outcomes of the child model group, this study found that only self-efficacy significantly increased (*P*<.001) after training. Social connectedness and outcome expectation also improved, but not at a significant level (*P*>.05)  In the young adult model group, again, only self-efficacy significantly increased after the training. Similar to the results in the child model group, social connectedness and outcome expectation improved slightly, but not at a significant level (*P*>.05).  In the older adult model group, both self-efficacy and social connectedness significantly increased after training. Outcome expectation improved, but not at a significant level (*P*>.05).  *Between groups findings:* Self-efficacy improvement after training was significantly different among the three groups (F=3.878; *P*<.05). In addition, recommendation willingness after training was also significantly different across the three groups (F=6.031; *P*<.01). No significant difference emerged with respect to the cognitive knowledge quiz scores of the participants across the three training groups (F=.029; *P*>.05).  For self-efficacy improvement, the older adult model group contributed to the highest improvement, and no significant difference between the child model group and the young adult model group emerged. As for recommendation willingness, no significant difference was found between the child model group and the older adult model group; however, both groups were significantly better, when compared with the young adult model group (*P*<.05). |  |
| Martínez-Alcalá et al. (2018). [Digital inclusion in older adults: A comparison between face-to-face and blended digital literacy workshops.](https://www.frontiersin.org/articles/10.3389/fict.2018.00021/full) *Frontiers in ICT,* 5, p.21.  Mexico | **Study Design:** Quasi-experimental study  **Type of intervention:** This study consisted of two groups both receiving an intervention. Workshops were delivered to groups of 15-25 older adults and lasted ∼ 4 months. They were carried out in computer rooms equipped with All-One computers connected to Internet. Every older adult was provided with a printed manual with information on the modules and topics addressed during the workshops. A tutor led every workshop, and they determined the topics to be developed.  *Face-to-face workshops*: Regarding learning methods, each student had a printed manual with the topics that would be studied in the workshop. For the instruction of digital skills, the tutor used digital presentations and a projector as support material.  *Blended workshops:* Learners had guides, activities, multimedia learning materials (digital presentations, videos, web pages) and resources that allowed them to acquire the necessary knowledge. Some materials could be viewed within the platform and others were distributed through links or they could even be downloaded for local reproduction on the equipment. In this workshop both the teacher and the student worked together to build knowledge, generate learning and develop digital skills more easily.  **Comparator:** Between-group comparisons were made as well as within group comparisons made pre- vs post-intervention  **Study aim:** To show a blended workshop based on a Learning Management System (LMS) as a supporting tool for older adults’ digital literacy.  **Data collection methods and dates:** A modified “Senior Digital Literacy Evaluation (SDLE)” instrument was used to assess digital literacy of participant before and after the intervention**.** Dates of data collection: Workshops started in 2014**.**  **Outcomes reported:**   - Digital competence and digital literacy - Perceived ease of use - Perceived usefulness - Attitude toward using - Intended use | **Sample size:** 98 participants (61 in face-to-face group and 37 in the blended workshop group)  **Participants:** Older adults (60 years and older)  **Setting:** Not stated, however, the interventions were carried out by The Academic Area of Gerontology at the Instituto de Ciencias de la salud (ICSa) of the Universidad Autónoma del Estado de Hidalgo. | **Primary Findings:** The workshops ameliorated the older adult digital competence in both groups (z=−6.79, *P*<.0001; z=−5.30, *P*<.0001, for the FFG and the BLG, respectively).  Participants in the blended workshop group reported a significantly greater post-intervention improvement in Senior Digital Literacy Evaluation (SDLE) scores compared to the face-to-face group (U(61, 37)=810.5, *P*<.01).  **Additional Findings:**  Perceived ease of use  In the analysis of the first variable, ease of use, 13 of the older adults indicated a positive agreement stating that the interaction with the system is clear and understandable and even the menu is easy to use.  Perceived usefulness  In the variable of perceived usefulness, favourable results were obtained from older adults, where 16 stated that it is useful and indispensable to implement this type of workshops so that the population acquires digital literacy skills.  Attitude toward using  The evaluation of attitude toward using showed that 15 older adults were enthusiastic about using the platform  Intended use  In the intention to use variable, 15 older adults indicated a positive agreement stating that they will use the system to reinforce their knowledge during and after the workshop | This study also measured participants experiences and perceptions of the blended workshop, however this data is non-comparative so not extracted. |
| Martínez-Alcalá et al. (2021). [The effects of Covid-19 on the digital literacy of the elderly: norms for digital inclusion.](https://www.frontiersin.org/articles/10.3389/feduc.2021.716025/full?trk=article-ssr-frontend-pulse_x-social-details_comments-action_comment-text) *In Frontiers in Education*(Vol. 6, p. 245). Frontiers.  Mexico | **Study Design:** Quasi-experimental/longitudinal study  **Type of intervention:** This study contains three different intervention modalities that occurred as the COVID-19 pandemic caused the digital inclusion service to adapt. The intervention moved from blended learning (pre-pandemic), to transition treatment (start of the pandemic) and then a fully digital intervention (during the pandemic once the service had adapted). The service offered multiple courses for different skill levels of participants. These course pathways were Basic 1 to Basic 3 (B1 to B3) and Intermediate 1 to Intermediate 3 (I1 to I3). Courses ran for 3 to 4 months and participants could progress through the courses to develop their digital literacy skills.  **Comparator:** Pre vs post intervention comparisons as well as comparisons between different treatments (e.g. blended learning vs transition treatment). Participants were divided into two groups (G1 and G2) based on their entry level digital literacy ability. G2 started the course at intermediate level and G1 at basic.  **Study aim:** To analyse the level of Digital Literacy with the Digital Literacy Evaluation (DILE) of two groups of elderly adults with different levels of literacy during three stages: Blended learning, Transition and Digital.  **Data collection methods and dates:** The “Digital Literacy Evaluation (DILE)” (formerly SDLE) tool was implemented pre- and post-intervention for participants when undertaking a course. The DILE is an instrument that was designed to measure the digital literacy level**.** Dates of the different treatments are as follows:   - Blended learning: Aug to Dec 2019 - Transition treatment: Feb-Jun 2020 - Digital treatment: Aug-Dec 2020   **Outcomes reported:**   - Digital literacy | **Sample size:** 20 participants were considered for longitudinal analyses and 251 for pooled analyses.  **Participants:** Older adults (aged around 60) living mainly in the state of Hidalgo and its surroundings.  **Setting:**   - Blended learning: community setting - Transition treatment and Digital treatment: remote | **Primary Findings:** *Longitudinal analysis (n=20):* for the comparison among the scores of the three pre-treatments and the three post-treatments of each course for each group, the three scores of the pre-treatments and the three scores for the post-treatments, were statistically significant.  Wilcoxon non-parametric tests were used as post-hoc tests and revealed differences for the G1 on the DILE scores over the pre-treatments in all cases: between the blended learning treatment and the next treatment before the pandemic was evident (z=−2.36, *P*=.01); between the DILE scores of the blended pre-treatment and that of the digital modality (z=−2.89, *P*=.003); and between the DILE scores for the transition to pandemic to the digital modality (z=−2.90, *P*=.003). For the post-treatments in the above-mentioned modalities, z=−2.75, *P*=.005; z=−2.98, *P*=.003; z=−2.89, *P*=.003, respectively. For the G2, in the pre-treatment condition and the above mentioned types of treatments, post-hoc tests between the DILE scores were, respectively: z=−.14, *P*=.88; z=−2.61, *P*=.009; z=−2.61, *P*=.009. For the post-treatments in the G2, DILE scores were, respectively for the blended to the transition to pandemic, z=−1.70, *P*=.08; z=−2.61, *P*=.009; z=−2.61, *P*=.009. Therefore, only in the G2, both the pre-treatment and the post-treatments were not statistically significant between the blended and the transition to pandemic, however the learners requested to repeat a course rather than progress to the next level so this may explain the non-significant change.  To further explore how the differences between groups were along treatments, differences between the post minus pre scores were calculated with a mixed ANOVA. Therefore, the two groups constituted the independent factor, and the repeated measure factor was the type of treatment (blended, transition to pandemic and digital). For the G1, the post minus pre differences within the semester for the blended treatment were 54.63 ± 14; for the transition to pandemic 43.90 ± 20; and for the digital treatment, 47.72 ± 17. For the G2, the post minus pre differences were: for the blended treatment 45.88 ± 7; for the transition to pandemic 49.11 ± 9; and for the digital treatment, 33.77 ± 5. Only the independent factor was significant (F=4.69, df=1,58; *P*=.04). The repeated measures factor was not statistically significant (F=1.85, df=2,58; *P*=.17) and there were not interaction effects (F=1.97, df=2,36; *P*=.15).  *Comparisons Within Different Treatments of the Same Digital Level:* Two extra comparisons using DILE scores were performed between modalities of treatments (blended or digital) within the same level (B1 or I1) in different groups and semesters. The first comparison comprising B1 was not significant (blended group from August to December, 2019 (n=69); pre: 128.37 ± 17; post: 184.59 ± 19; digital group from August to December, 2020 (n=41): pre: 132.39 ± 23; post: 194.19 ± 30; for the pre-pre comparison, t=0.96, *P*=.33; for the post-post comparison, t=1.79, *P*=.07). Instead, for the intermediate level, I1, the digital group was certainly more digitally literate than the blended one both for the pre and the post comparisons (digital group from August to December, 2020 (n=11); pre: 215.18 ± 12; post: 262.91 ± 18; blended group from August to December, 2019 (n=13): pre: 199.08 ± 13; post: 246.85 ± 14; for the pre-pre comparison, t=2.99, *P*=.006; for the post-post comparison, t=2.36, *P*=.02).  **Additional Findings:**  *Dropouts:* There was a great rate of dropouts before the pandemic occurred. For the first group, the percentage of the dropout–from the blended course (Aug Dec 2019) to the beginning of the transition to pandemic course (Feb June 2020) had been of 57.97% (G1: n=69 for the first semester and n=29 for the second semester). For the second group, the dropout was 23.52% (G2: n=17 for the first semester and n=13 for the second semester). The dropouts of the courses for the subjects that at least had assisted continuously during two semesters from the blended to the transition to pandemic- and then, withdrew from the digital course were obtained. The dropout between these periods were, for each group, 62.0 and 30.76%, respectively (G1: n=29 for the first semester, n=29 for the second semester and n=11 for the third semester; G2: n=13 for the first semester, n=13 for the second semester and n=9 for the third semester). When only the semester of the pandemic is considered, a great dropout occurred especially for B1: only five older adults could complete the entire course and filled the pre and post DILEs, and 19 older adults abandoned the course (they only filled the DILE on the pre-treatment), therefore, the dropout reached 79.16%. | This intervention assesses the effects of the COVID-19 pandemic, specifically the transition of digital literacy services from face-to-face to fully digital, on digital literacy.  A small number of participants (n=20) were included for longitudinal analysis  Pooled analysis was conducted for the whole sample (n=251) however statistical comparisons were not performed  This study includes participants who already possessed digital literacy skills as well as those with limited skills. |
| McCosker et al. (2023). Accounting for diversity in older adults’ digital inclusion and literacy: the impact of a national intervention. Ageing & Society, pp.1-21.  Australia | **Study Design:** Quasi experimental (mixed methods)  **Intervention:** A national digital inclusion programme (Be Connected) with the stated aim of improving the confidence, skills and online safety of people aged 50 years and over. There are two core components to the programme’s design: online learning modules presented through a government Web portal, and face-to-face support provided by a network of community-based organisations.  **Comparator:** Pre vs post intervention comparisons  **Data collection methods and dates:** Data were collected between Aug 2018 (1^st^ wave) and May 2019. (2^nd^ wave). This study used a mixed methods approach that involved a pre- and post- survey with semi structured interviews (N=58) with learners (N=38) and digital mentors (N=20) conducted in person or by phone to accommodate geographical diversity. The survey instrument was designed to combine measures of participants’ online confidence, attitudes, digital skills and digital activities in a way that would provide holistic indicators of digital participation, and pinpoint key aspects of situated digital literacies.  **Outcomes reported:** Surveys included outcomes related to:   - Changes to operational and strategic skills - Changes to digital confidence - Changes to digital participation | **Sample size:** 337  **Participants:** Older adults age 50 to 94 years  **Setting:** Online and face-to-face, community based | **Primary findings:**  *Changes to operational and strategic skills:* participants significantly improved ten operational and strategic skills post-intervention. This included positive changes to their ability to: install apps, keep track of mobile app costs, using shortcuts, saving photos and more. No significant change was found for ten skills including sharing information online, and keyword searching on websites.  *Changes to digital confidence:* participants significantly improved their digital confidence in 5/12 areas including, using email, being safe online, using search engines and using a smartphone or tablet. No significant improvement was found for 7/12 areas such as, using a computer, buying and selling things online, using social media, online banking, video calling and streaming music or TV.  *Changes to digital participation:* One out of 21 outcomes showed a significant post-intervention for digital participation changes. Watching online videos/TV programs increased, however no other significant improvements were evidenced. | Data from related paper from same author (McCosker et al (2020). [Improving the digital inclusion of older Australians: the social impact of Be Connected.](https://apo.org.au/sites/default/files/resource-files/2021-01/apo-nid305919.pdf)  Swinburne University) has been utilised for this review. See McCosker (2020) appendix B for full results table. |
| Moore and Hancock (2022). [A digital media literacy intervention for older adults improves resilience to fake news](https://www.nature.com/articles/s41598-022-08437-0). *Scientific reports,* 12(1), p.6008.  USA | **Study Design:** Controlled before and after study  **Intervention:** The MediaWise for Seniors intervention - a self-directed online course which taught digital media literacy skills and techniques helpful for verifying the credibility of information online, such as lateral reading and reverse image searching  **Comparator:** No intervention  **Study aim:** To examine the effect of a digital literacy intervention in improving older adults’ resilience to fake news  **Data collection method and dates:** Data were collected via the use of pre- and post-course surveys. No dates were provided, however intervention group participants were recruited from September 24 to December 2, 2020, while control group participants were recruited from October 1 to November 6, 2020  **Outcomes reported:**   - Deception detection - Comprehension and use of digital media literacy techniques | **Sample size:** 381 participants (143 in intervention and 238 in the control group)  **Participants:**  Intervention group: older adults (mean age 67.2 years, 67.4% female, 86.8% White) completed the MediaWise for Seniors course (intervention) and both the pre- and post-course surveys.  Control group: older adults (Mean age = 63.8 years, 60.5% female, 89.5% white) recruited from online survey purveyor Lucid also completed both the pre- and post surveys, without taking MediaWise’s course or being exposed to any other control stimuli  **Setting:** Online | **Primary Findings:** There was a significant improvement in the ability to accurately judge the veracity of news headlines among the intervention group from pre-intervention to post-intervention compared to the control group (*P*<.001). Older adults in the treatment condition (N=143) significantly improved their likelihood of accurately discerning fake from true news from 64% (95% CI: 61 to 67%) pre-intervention to 85% (95% CI: 82 to 88%) post-intervention. In contrast, older adults in the control condition (N=238) did not significantly improve - from 55% (95% CI: 53 to 58%) pre-intervention to 57% (95% CI: 55 to 60%).  Older adults in the treatment condition showed significantly greater understanding and use of skills and techniques important for identifying misinformation online after the course compared to before. There was a positive and significant interaction between the Intervention Group and Post-intervention variables (B=4.436, SE=.505, *P*<.001), indicating that the increase in the likelihood of reporting doing research to inform a headline veracity judgment among the intervention group from pre-intervention to post-intervention was significantly greater than change in the likelihood of doing research among the control group. The intervention group rose from a pre-intervention probability of doing research to inform their headline judgments of 4% (95% CI: 1 to 6%) to a post-intervention probability of 71% (95% CI: 64 to 78%). This pattern was not observed in the control group (their pre-intervention researching probability=3% (95% CI: 1 to 4%), post-intervention probability=2% (95% CI: 1 to 3%). | The focus of this study was on improving the ability of older adults to detect misinformation online. However, this was done by the use of a digital media literacy intervention. |
| Ngiam et al. (2022). [Building Digital Literacy in Older Adults of Low Socioeconomic Status in Singapore (Project Wire Up): Nonrandomized Controlled Trial](https://www.jmir.org/2022/12/e40341/). *Journal of Medical Internet Research,* 24(12), p.e40341.  Singapore | **Study Design:** Non-randomised controlled study  **Intervention:** Project Wire Up, a  volunteer-led, one-on-one, and home-based digital literacy programme.  **Comparator:** Older adults awaiting participation in the programme (i.e., no intervention)  **Study aim:** To examine the impact of a volunteer-led, one-on-one, and home-based digital literacy programme on  digital literacy and health-related outcomes such as self-reported loneliness, social connectedness, quality of life, and well-being for older adults of low socio-economic status.  **Data collection method and dates:** Data were collected from participants either in person or via telephone through standardised self-reported questionnaires in participants’ preferred language. Participants recruited from July 2020 to November 2020 were assigned to the intervention arm, whereas participants recruited from November 2020 to July 2021 were assigned to the control arm. Data collection was completed in November 2021.  **Outcomes reported:**  Primary outcome   - Digital literacy score (To measure digital literacy, a 13-item self-reported digital literacy scale was constructed based on 4 domains of smartphone usage)   Secondary outcomes   - Lubben Social Network Scale-6 (LSNS-6) - University of California, Los Angeles 3-item loneliness scale (UCLA-3) - EQ-5D - Personal Wellbeing Score (PWS) | **Sample size:** 138 participants  **Participants:** Digitally excluded community-dwelling older adults (55 and older) of lower socio-economic status residing in Singapore  **Setting:** Home-based | **Primary Findings:** The intervention group observed a statistically significant difference in the change in their mean digital literacy score before and after programme, as compared to those in the control group (mean difference: 2.28, 95% CI: 1.37 to 3.20; *P*<.001).  Through multiple linear regression analyses, this change in digital literacy scores remained independently associated with group membership after adjusting for baseline digital literacy scores and differences in age, gender, education, living arrangement, housing type and baseline social connectivity and loneliness status (Model 2, β=1.91, 95% CI: .93 to 2.89; *P*<.001 and Model 3, β=1.90,95% CI: .91 to 2.90, *P*<.001).  The domain-level analyses showed that the greatest gain was in the Instrumental domain (obtaining news and information and accessing health, government, and banking services), where the participants in the intervention arm learned, on average, approximately 1 more new function than the control arm, followed by the Reassurance, Social, and Pastime domains. The before and after program difference in all domains except for the Pastime domain remained statistically significant after controlling for covariates. | Participants were assigned  to either the intervention or control arm using convenience sampling based on the referral timing to the programme.  In this study, digital literacy was defined as the knowledge of the functional use of smartphones |
| Patty et al. (2018). [A cost-effectiveness study of ICT training among the visually impaired in the Netherlands](https://bmcophthalmol.biomedcentral.com/articles/10.1186/s12886-018-0761-y). *BMC ophthalmology,* 18(1), pp.1-10.  The Netherlands | **Study Design:** Before and after study  **Intervention:** Information and communication technology (ICT) training (this included computer training and training sessions on the use of iPhones, iPads and digital assistant devices)  **Comparator:** No intervention (pre-post)  **Study aim:** To assess the cost-effectiveness of ICT training among visually impaired adults from a societal perspective, using primary data from two large rehabilitative eye care providers in the Netherlands.  **Data collection method and dates:** Data were collected using pre – and post – training questionnaires. No dates were provided, however, the ICT training was conducted between July 2014 and January 2015  **Outcomes reported:**   - Health-related quality of life and well-being (measured using the EQ-5D and the ICECAP-O) - Healthcare consumption and productivity losses - measured using the Medical Consumption Questionnaire (iMCQ) and the Productivity Cost Questionnaire (iPCQ) respectively. - Care-related Quality of Life and rehabilitation needs of visually-impaired persons – measured using the CarerQol and an adapted version of the Dutch Activity Inventory (D-AI) respectively. - Cost-effectiveness | **Sample size:** 45 participants  **Participants:** Visually impaired clients of two large rehabilitative eye care providers in the  Netherlands who were enrolled in ICT training between July 2014 and January 2015 (mean age 63).  **Setting:** Two large rehabilitative eye care providers in the Netherlands. | **Primary Findings:** Of the 45 participants who completed the questionnaire pre - training and post - training, 58% (26) were women. The mean age was 63 years (range 27–90 years).  ***Impact of the ICT training***  The effect of ICT training on ICT skills and participants’ well-being was positive and persisted three months after the last training session. The health-related quality of life measured with the EQ-5D improved slightly after the ICT training, from .70 to .73. Furthermore, an increase in well-being (ICECAP-O) was observed immediately after the training, with a mean score of .81 compared to .77 before the training.  The mean D-AI score (ICT skills) decreased from the initial outcome by 9.9 points, to 13.1, indicating a positive effect on ICT skills. The most noteworthy changes were observed in the areas of computer skills, the Internet and use of hotkeys.  ***Cost-effectiveness***  ICT training appears to be cost-effective under the assumption that the effects of ICT training on well-being remain constant for five or 10 years.  Assuming these effects remain constant for 10 years, this would result in an incremental cost-effectiveness ratio (ICER) of € 11,000 per quality-adjusted life-year (QALY) and € 8000 per year of well-being gained, when only the costs of ICT training are considered. When the total costs of medical consumption are included, the ICER increases to € 17,000 per QALY gained and € 12,000 per year of well-being gained. Furthermore, when the willingness-to-pay threshold is € 20,000 per year of well-being, the probability that ICT training will be cost-effective is 75% (91% when including only the costs of ICT training). | This study was targeted at visually impaired individuals, however, during the course of the study, it was noted that the majority of participants were elderly (60 and older). Adjustments were then made to the outcome measures and instruments so that these were specifically aimed at measuring outcomes in the elderly.  The study authors stated that as the ICT training was a part of standard rehabilitative care, the use of a control group without such training was considered impossible and  unethical. |
| Quialheiro et al. (2023). [Promoting Digital Proficiency and Health Literacy in Middle-aged and Older Adults Through Mobile Devices With the Workshops for Online Technological Inclusion (OITO) Project: Experimental Study](https://formative.jmir.org/2023/1/e41873). *JMIR Formative Research,* 7, p.e41873.  Portugal | **Study Design:** Non-randomised Quasi-experimental study design  **Intervention:** The OITO (Oficinas de Inclusão Tecnológica Online, “Workshops for Online Technological Inclusion”) project.  **Comparator:** No intervention (pre-post)  **Study aim:** To develop, conduct, and measure the impact, on digital and health literacy, of an in-person 8-workshop guided digital inclusion project  aimed at community dwellers aged 55 years or older in 3 northern Portuguese cities.  **Data collection method and dates:** Data were collected using questionnaires at baseline (T1), immediately after completing the intervention (T2), and at 1 month after the intervention (T3). The intervention took place between May 2021 and January 2022.  **Outcomes reported:**  Primary outcome   - Digital literacy – measured using the 16-question Mobile Device Proficiency Questionnaire (MDPQ-16)   Secondary outcome   - Health literacy – measured by the 12-question Health Literacy Scale (HLS-12)   Self-reported autonomy | **Sample size:** 87  **Participants:** People aged 55 and older that owned mobile devices with an internet connection in 3 cities in northern Portugal  **Setting:** Not specified but data were collected at workshops locations varied based on specific project partners. | **Primary Findings:**  At baseline, participants had low baseline scores in digital literacy, but medium-high baseline scores in health literacy. Analysis over time indicated a significant improvement in digital literacy, both immediately after the conclusion of the workshops (T2) and 1 month afterward (T3) compared to baseline (T1), but without significant differences in digital or health literacy scores between the post-workshop times (T2 and T3), regardless of sex, age, or schooling.  A significant improvement in self-reported autonomy was observed at T3 compared with baseline, increasing from 4.5 to 6.7 points, with a score range from 0 to 10 (t40=–7.3; *P*<.001).  In longitudinal analysis of digital literacy over time, adjusted by sex, age, and education level, both women and men showed positive growth in both types of literacy. All age categories showed improved post-intervention scores, and the overall change in the performance of the participants was similar for all education levels. There was an average increase of 2.49 points in the digital literacy score in the raw analysis and an average increase of 2.46 points in the analysis adjusted for sex, age, and education level. None of these variables revealed a significant interaction with time, meaning that the different subgroups varied similarly across the 3 time points. | A convenience sample of participants was recruited for this study |
| Seaton et al. (2023). [Gluu Essentials Digital Skills Training for Middle-Aged and Older Adults That Makes Skills Stick: Results of a Pre-Post Intervention Study](https://aging.jmir.org/2023/1/e50345/). *JMIR aging,* 6(1), p.e50345.  Canada | **Study Design:** Before and after study (Pre-post cross-sectional survey design)  **Intervention:** The Gluu Essentials digital skills training program. A digital skills training programme developed by Gluu Society and delivered in partnership with local community organisations and volunteer coaches.  **Comparator:** No intervention (pre-post)  **Study aim:** To examine the effectiveness and programme acceptability of a digital skills training programme among middle-aged and older adults (aged ≥50 years) and to gather participants’ recommendations for lifelong digital skills promotion.  **Data collection method and dates:** Data were collected using pre – and post – test questionnaires. No dates provided, however surveys were completed in 2021 and 2022.  **Outcomes reported:**   - Self-report measures of mobile device proficiency and confidence - Mobile Device Proficiency Questionnaire (MDPQ), and the frequency of engaging in online activities were collected at both baseline and follow-up.   Programme engagement, acceptability, and suggestions were completed at follow-up only. | **Sample size:** 264 participants (145 completed the follow up survey)  **Participants:** Middle-aged and older adults (50 and older) in rural and urban communities in Canada  **Setting:** Sessions were mainly provided from a distance (self-directed with telephone support) however some organisations delivered in-person support in small groups | **Primary Findings:** Mobile device proficiency improved significantly from baseline to follow-up: MDPQ total score =3.93 (SD=.91) – 4.13 (SD=.79); *P*<.001. Confidence in going online (*P*=.66) and in avoiding frauds and scams (*P*=.54) did not change significantly from baseline to follow-up.  The comparison of the groups showed the frequency of going online for shopping (*P*=.01) and accessing government services (*P*=.02) increased, whereas the frequency of going online for email (*P*=.47), banking (*P*=.10), information (*P*=.96), and emergency services (*P*=.42) did not change significantly. The frequency of going online for COVID-19–related information significantly decreased (*P*=.01).  **Additional Findings:**  Programme engagement varied considerably, but programme acceptability was high. Participants’ recommendations included the need for providing ongoing programmes for support and training because technology constantly changes, reducing costs for technology and internet access, and keeping learning resources simple and easy to access. | Although this study included participants aged 50 years and older, the mean age of the participants was 72.93 years. |
